# Metagenomic Analysis of Viral Communities in (Hado)Pelagic Sediments

**DOI:** 10.1371/journal.pone.0057271

**Published:** 2013-02-27

**Authors:** Mitsuhiro Yoshida, Yoshihiro Takaki, Masamitsu Eitoku, Takuro Nunoura, Ken Takai

**Affiliations:** Japan Agency for Marine-Earth Science and Technology (JAMSTEC), Yokosuka, Kanagawa, Japan; University of Kansas Medical Center, United States of America

## Abstract

In this study, we analyzed viral metagenomes (viromes) in the sedimentary habitats of three geographically and geologically distinct (hado)pelagic environments in the northwest Pacific; the Izu-Ogasawara Trench (water depth = 9,760 m) (OG), the Challenger Deep in the Mariana Trench (10,325 m) (MA), and the forearc basin off the Shimokita Peninsula (1,181 m) (SH). Virus abundance ranged from 10^6^ to 10^11^ viruses/cm^3^ of sediments (down to 30 cm below the seafloor [cmbsf]). We recovered viral DNA assemblages (viromes) from the (hado)pelagic sediment samples and obtained a total of 37,458, 39,882, and 70,882 sequence reads by 454 GS FLX Titanium pyrosequencing from the virome libraries of the OG, MA, and SH (hado)pelagic sediments, respectively. Only 24−30% of the sequence reads from each virome library exhibited significant similarities to the sequences deposited in the public nr protein database (E-value <10^−3^ in BLAST). Among the sequences identified as potential viral genes based on the BLAST search, 95−99% of the sequence reads in each library were related to genes from single-stranded DNA (ssDNA) viral families, including *Microviridae*, *Circoviridae*, and *Geminiviridae*. A relatively high abundance of sequences related to the genetic markers (major capsid protein [VP1] and replication protein [Rep]) of two ssDNA viral groups were also detected in these libraries, thereby revealing a high genotypic diversity of their viruses (833 genotypes for VP1 and 2,551 genotypes for Rep). A majority of the viral genes predicted from each library were classified into three ssDNA viral protein categories: Rep, VP1, and minor capsid protein. The deep-sea sedimentary viromes were distinct from the viromes obtained from the oceanic and fresh waters and marine eukaryotes, and thus, deep-sea sediments harbor novel viromes, including previously unidentified ssDNA viruses.

## Introduction

Viruses represent the most abundant number of biological components by far in aquatic ecosystems [1], and viral ecology in environments such as oceanic surface waters, coastal, and fresh waters have been intensively investigated [2]. Viral activity in aquatic environments is known to regulate the dynamics and mortality of the host microbial community [3]–[9]. The lytic processes of the host microbial cells infected by marine viruses, termed the “viral shunt”, supply organic matter to dissolved carbon and nutrient pools [10]–[12]. Furthermore, viruses have been noted as natural genetic vectors for horizontal gene transfer events [13], [14]. Despite their ecological and evolutionary importance, our current knowledge of marine viruses is restricted to the euphotic zone of the habitat, which represents only a limited portion of the oceanic biosphere [15]. Viral ecology in sedimentary environments has been poorly studied, although the seafloor sediments cover almost two-thirds of the Earth’s surface and serve as highly vital and dynamic interface habitats in global ocean biogeochemical cycles [16].

Deep-sea sediments (down to 10 cm below the seafloor [cmbsf]) harbor a great number of viral particles (>10^7^ viruses/cm^3^ sediment) and high virus productivity associated with large prokaryotic biomasses ranging from 10^6^ to 10^8^ cells/cm^3^ sediment [17], [18]. These observations suggest that viral infections have a large impact on deep-sea sedimentary microbial communities and that the benthic prokaryotic biomass is sustained by the “viral shunt”, which is estimated to provide 35% of organic carbon for the total benthic microbial production [18]. However, the genetic composition and diversity of viral communities in deep-sea sediments have not yet been reported.

A comprehensive metagenomic approach to environmental viral populations (viromes) can provide insight into the genetic diversity and previously unidentified constituents of the viral communities of various ecosystems [19]–[26]. Two different whole-genome amplification methods have been used for virome analysis. One is known as the linker-amplified shotgun library (LASL) method [27] and is only applicable to double-stranded DNA (dsDNA). The LASL method has been applied in several virome studies, such as of surface seawater [27], human feces [28], and fermented foods [29]. These virome studies have suggested that a large proportion of the DNA viruses infect prokaryotic hosts, while most RNA viruses analyzed by reverse transcription infect eukaryotes [30]. The other method, known as multiple displacement amplification (MDA) with phi29 polymerase [31], can amplify both the dsDNA and single-stranded DNA (ssDNA) of the viral genomes, although this method is known to have a considerable bias for the preferential amplification of small circular genomes (1−9 kb) from ssDNA viruses [32], [33]. Using this method, the distribution and diversity of ssDNA viruses (including both phages and eukaryotic viruses) have been investigated in various environments, such as marine waters [19], [34], modern microbialites [35], coral [36], temperate freshwater lakes [37], the Antarctic lake [21], reclaimed water [38], the human gut [39], [40], and rice paddy soil [33]. However, the host ranges and ecological impacts of these ssDNA viruses are still largely uncertain [41].

In this study, we used 454 pyrosequencing to conduct a virome analysis of deep-sea shallow subseafloor sediments (down to 40 cmbsf) in three distinct (hado)pelagic environments of the northwest Pacific: the hadopelagic sediments in the Izu-Ogasawara Trench (water depth = 9,760 m), the hadopelagic sediments in the Challenger Deep of the Mariana Trench (water depth = 10,325 m), and the pelagic sediments off Shimokita Peninsula (water depth = 1,181 m). To our knowledge, this study is the first to describe the characteristics of viromes in deep-sea sediments and identify novel ssDNA viruses that are distinct from viral genotypes previously known to occur in ocean environments.

## Materials and Methods

### Ethics Statement

The sampling in the Mariana Trench during the JAMSTEC KR08-05 cruise was approved by the U.S. Government. No specific permits were required for the other field studies described here and sampling locations are not privately-owned or protected. The field studies did not involve endangered or protected species.

### Sediment Samples

**Figure 1:**
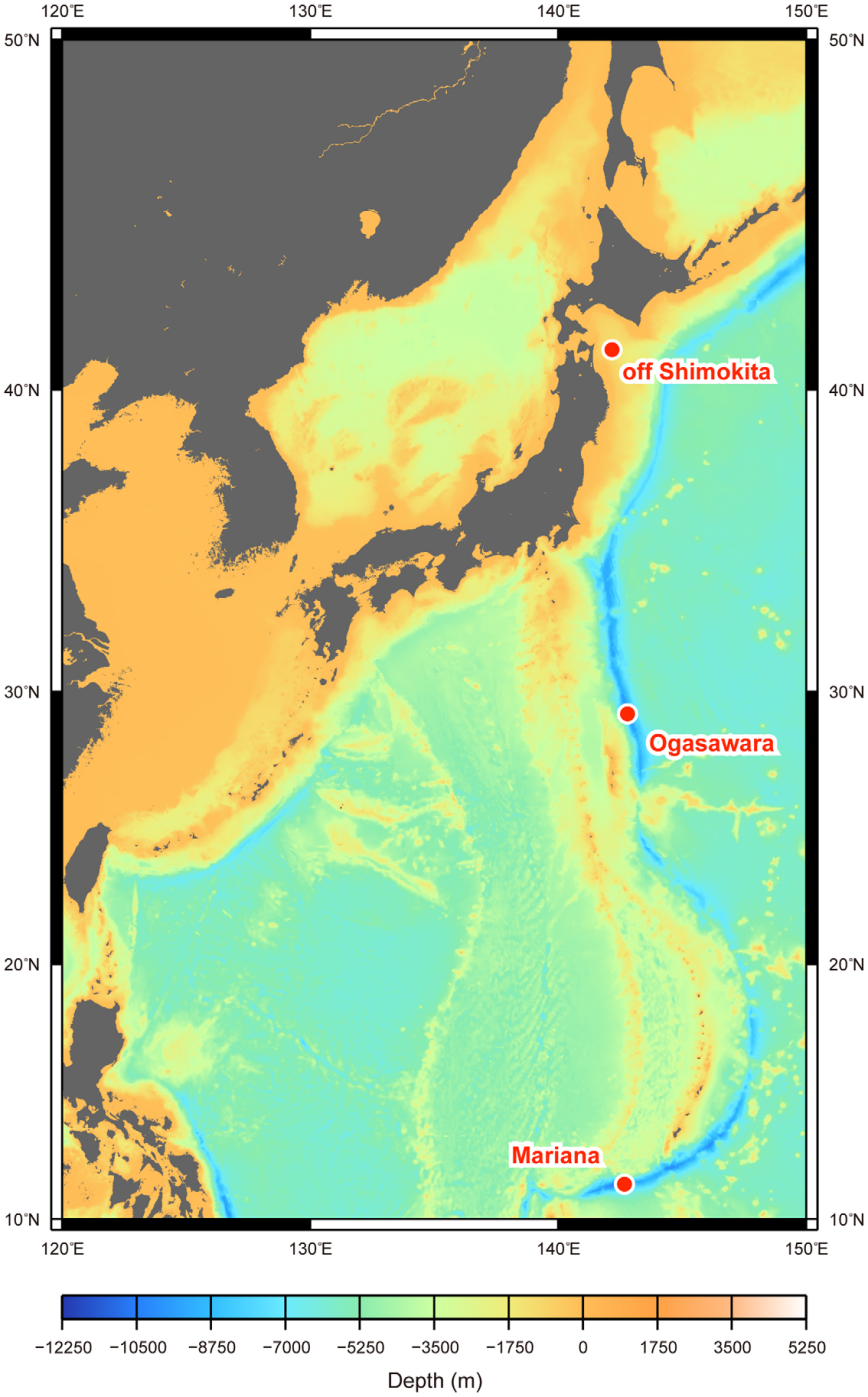
Sampling location of the (hado)pelagic sediments used in this study.

Sediment cores from the Izu-Ogasawara Trench (29°09′ N, 142°49' E; 9,760 m water depth) (Fig. 1) and the Challenger Deep in the Mariana Trench (11°22′ N, 142°42′ E; 10,332 m water depth) (Fig. 1) were obtained with a gravity corer of the ROV *ABISMO* (Automatic Bottom Inspection and Sampling Mobile) during the JAMSTEC KR07-17 (December 2007) and KR08-05 (May−June 2008) cruises with the R/V *Kairei* [42], respectively. The lengths of the cores were 1.0 m and 1.3 m from the Izu-Ogasawara and Mariana Trench, respectively. A short core (40 cm in length) of seafloor surface sediment from offshore of the Shimokita Peninsula was obtained using a push corer of the ROV *HyperDolphin* during the JAMSTEC NT06-13 cruise (Dive #581∶41°10′ N, 142°12′ N; 1,181 m water depth) (Fig. 1) with the R/V *Natsushima*. Each sediment core was subsampled from top to bottom at every 2−10 cm interval using sterilized top-cut 50 mL syringes or spatulas. Subsamples were stored at −80°C until the viral DNA was collected. The total organic carbon of each subsample was estimated with a Flash EA1112 elemental analyzer (Thermo Fisher Scientific, Waltham, MA, USA) at S1 Science (Saitama, Japan).

### Prokaryotic 16S rRNA Gene Clone Analyses

To identify the phylotype compositions of the prokaryotic communities in the (hado)pelagic surface sediments, DNA was extracted with a PowerSoil DNA Isolation kit (Mo Bio Laboratories, Carlsbad, CA, USA) following the manufacturer’s instructions. DNA was extracted from approximately 0.25 g of sediments that were a portion of the same subsample used for the virome analysis. The method for this clone library analysis is described in the supplementary material (Materials and Methods S1).

### Direct Counting of Viral Particles

To enumerate the viral particles, approximately 1 cm^3^ of frozen sediment was promptly suspended in 10 mL of modified SM buffer (10 mM MgSO_4_; 50 mM Tris-HCl, pH 7.5) containing 3% NaCl (w/v) and 2% formaldehyde in a 50 mL centrifuge tube. The slurry was shaken with a ShakeMaster (BioMedical Science, Tokyo, Japan) for 1 min at the maximum speed and then sonicated for 1 min with an ultrasonic homogenizer (UH-50; SMT company, Tokyo, Japan) to detach viruses from sediment matrices [43]. After centrifugation, the size fraction of prokaryotes was removed from the supernatants through a 0.2 µm cut-off filter. The viral population was then filtered onto a 0.02 µm pore-size Anodisc membrane filter (Whatman, Kent, UK). The filters were rinsed thoroughly three times with 2 mL of fresh SM buffer, and the viruses on the filter were stained with 20× SYBR Gold (Invitrogen, Carlsbad, CA, USA) at room temperature for 20 min [44]. After rinsing with pure water, each filter was mounted on a glass slide with immersion oil. Viruses on the filter were observed with a fluorescence microscope (model BX61; Olympus, Tokyo, Japan) using a fluorescence filter set (WIB; Olympus). The number of viruses was counted in at least 10 microscopic fields for each sample.

### Construction of Virome Libraries

Sediment core samples at a core depth of 20−30, 0−10, and 5−10 cmbsf in the Ogasawara Trench, the Mariana Trench, and offshore the Shimokita Peninsula, respectively, were used to construct the libraries of viral metagenomes (viromes). The libraries were obtained by following the procedures described by Casas and Rhower [45] with minor modifications. A total of approximately 100 cm^3^ frozen sediments was suspended in 400 mL of modified SM buffer containing 3% NaCl (w/v), dispensed into 50 mL centrifuge tubes, and incubated for 1 h at 4°C. The slurry was shaken for 1 min with a ShakeMaster (BioMedical Science) at the maximum speed and centrifuged at 6,000×*g* for 15 min. The supernatant was filtered with a 0.2 µm filter, and viral particles were precipitated with 10% polyethylene glycol (PEG) 8000 (w/v) overnight at 4°C. Viral particles were collected by centrifugation at 11,000×*g* for 30 min. The viral fractions were further purified by cesium chloride (CsCl) density centrifugation as described previously [45]. The virome library was then obtained by using formamide and CTAB/NaCl according to Casas and Rhower [45]. The obtained libraries were amplified with a REPLI-g Midi Kit (Qiagen), and remnant ssDNA in the amplified genomes was digested with S1 nuclease (Invitrogen, Carlsbad, CA, USA).

### Virome Composition Analysis

The virome libraries from the deep-sea shallow subseafloor sediments were analyzed with a 454 GS FLX Titanium pyrosequencer (Roche, Basel, Switzerland) by Beckman Coulter Genomics (Danvers, MA, USA). An eighth of a PicoTiterPlate device was used to sequence each of the three virome libraries. The CLC Genomics Workbench ver. 5.5.1 (CLC Bio, Aarhus, Denmark) was used to remove poor quality reads (the parts with Phred quality scores lower than 20 were trimmed off; the rest of the trimmed reads have a length shorter than 100 bp) or artificial duplicates (they share a common sequence of at least 20 bp in the beginning; the rest of the reads have an alignment scores above 80% of the optimal score) and to assemble the trimmed reads from each library. The default values were used for all the parameters in the assembly. The obtained sequences of contigs and singletons were subjected to BLASTx analyses against the NCBI GenBank nonredundant (nr) protein database [46]. MEGAN (MEtaGenome Analyzer; version 4.61.6) software was used to assign taxonomic groups of viruses and cellular organisms (bacteria, archaea, and eukaryotes) to the sequences with significant BLAST hits (E-values >10^−3^) in the three libraries [47], [48]. The MEGAN-based taxonomic assignment was performed based on the top 10% of the significant hits.

### Functional Analysis of Virome Genes

Predicted protein-encoding sequences (CDSs) from the contigs in the virome libraries were identified with the MetaGeneMark [49] and Glimmer-MG [50] programs, and additional CDSs were identified by BLASTx searches. In the partially overlapping CDSs from two different methods, the longer one was used for the analysis. These full and partial CDSs were classified functionally according to the SEED-subsystems [51] based on the BLASTp search results. The top 10% of the significant hits (E-value <10^−3^) were used to infer gene functions.

### Phylogenetic Analysis of the ssDNA Viral Genes

Two ssDNA viral markers (the major capsid protein [VP1] gene and the putative replication associated protein [Rep] gene) were used to construct the phylogenetic trees. These markers from the virome genes were screened based on significant sequence similarity (E-value <10^−3^ in BLASTp) to the references in the GenBank nr protein database and presence of the conserved Pfam domains (Pfam 26.0; http://pfam.sanger.ac.uk/): the Phage_F (PF02305) domain, in the VP1 genes; the Viral Rep (PF02407) or Gemini_AL1 (PF00799) domains, in the Rep genes. Multiple sequence alignments of the conserved domains in their marker genes were constructed by the MAFFT program [52], [53]. Phylogenetic analyses with the neighbor-joining method [54] were performed with the MEGA5.05 program [55].

### Comparison of Viromes

The virome libraries from the deep-sea shallow subseafloor sediments were compared with the virome data from other environments with MetaVir (http://metavir-meb.univ-bpclermont.fr/) [56]. In the MetaVir workflow, viromes were compared based on sequence similarity with a cross-tBLASTx search as described in Martín-Cuadrado et al. [57]. The viromes in deep-sea sediments were compared with all deposited viromes in the MetaVir using tBLASTx. A similarity score between the two viromes was computed as the sum of best BLAST hit scores of a sequence component in one virome library against a counterpart in the other virome library. Finally, the resulting score matrix (i.e., similarity scores for all virome pairs) was used to cluster the viromes with R software (version 2.14.0; http://www.r-project.org/) [58] and the PVCLUST package (http://www.is.titech.ac.jp/~shimo/prog/pvclust/) [59] using a construction method based on the unweighted pair-group method with arithmetic averages (UPGMA). The confidence of the clustering was assessed with the multiscale bootstrap resampling clustering algorithm in PVCLUST [59] and indicated by the approximate unbiased bootstrap probability at selected nodes.

### Nucleotide Sequence Accession Numbers

All pyrosequencing read data from the three virome libraries obtained in this study have been submitted to the DDBJ Sequence Read Archive (DRA) (http://trace.ddbj.nig.ac.jp/dra/index_e.shtml) under the accession number DRA000564. The sequences of the VP1 and Rep genes from the virome libraries used for the phylogenetic analyses of the ssDNA viral assemblages were deposited into the DDBJ/EMBL/GenBank nucleotide sequence databases under the accession numbers BAKA01000001 to BAKA01000006 (Ogasawara library), BAKB01000001 to BAKB01000011 (Mariana library), and BAKC01000001-BAKC01000114 (Shimokita library). The 16S rRNA gene sequences obtained in this study were deposited in the DDBJ/EMBL/GenBank nucleotide sequence databases under the accession numbers AB734482 to AB734640.

## Results

### Sample Characteristics

The deep-sea sediments used in this study were obtained from three geographically and geologically distinct areas in the Northwest Pacific (Fig. 1). The Challenger Deep in the Mariana Trench is the deepest part of the world’s oceans under the oligotrophic water masses [60]. The forearc basin off the Shimokita Peninsula is located in the area near the coast of northeastern Japan, with high primary production, and the sediments of the area are characterized by a large amount of subseafloor microbial biomass [61]. The sampling station at the Izu-Ogasawara Trench is one of the deepest points of the trench system. The shallowest sediment (down to 30 cmbsf) off Shimokita Peninsula contained high organic carbon contents (2.62−3.81% weight of TOC) compared with the shallow subseafloor sediments at the Ogasawara Trench and Mariana Trench (0.67−0.86 and 0.15−0.23 wt%, respectively), reflecting different oceanographic backgrounds between the (hado)pelagic sedimentary habitats [62].

The abundance of viruses in the shallowest 30 cmbsf sediments was determined to be 9.9×10^7^−1.8×10^11^ viruses/cm^3^ sediment in the off Shimokita Peninsula (SH) sediments, 5.8×10^7^−6.6×10^7^ viruses/cm^3^ in the Ogasawara Trench (OG) sediments, and 2.4×10^6^−5.3×10^7^ viruses/cm^3^ in the Mariana Trench (MA) sediments (Table S2). The abundance of viruses in the SH and MA decreased with increasing sediment depth but did not decrease significantly in the OG sediments. Based on the virus abundance data in the shallow subseafloor sediments, we chose subsamples of the sediments for the subsequent virome analysis and prokaryotic 16S rRNA gene clone analysis. The sediment samples with relatively high virus abundances of 7.6×10^10^ viruses/cm^3^ at a depth of 5−10 cmbsf in the SH sediments, 6.6×10^7^ viruses/cm^3^ at a depth of 20−30 cmbsf in the OG sediments, and 1.2×10^7^−5.3×10^7^ viruses/cm^3^ at a depth of 0−10 cmbsf in the MA sediments were used for the subsequent investigations.

**Figure 2:**
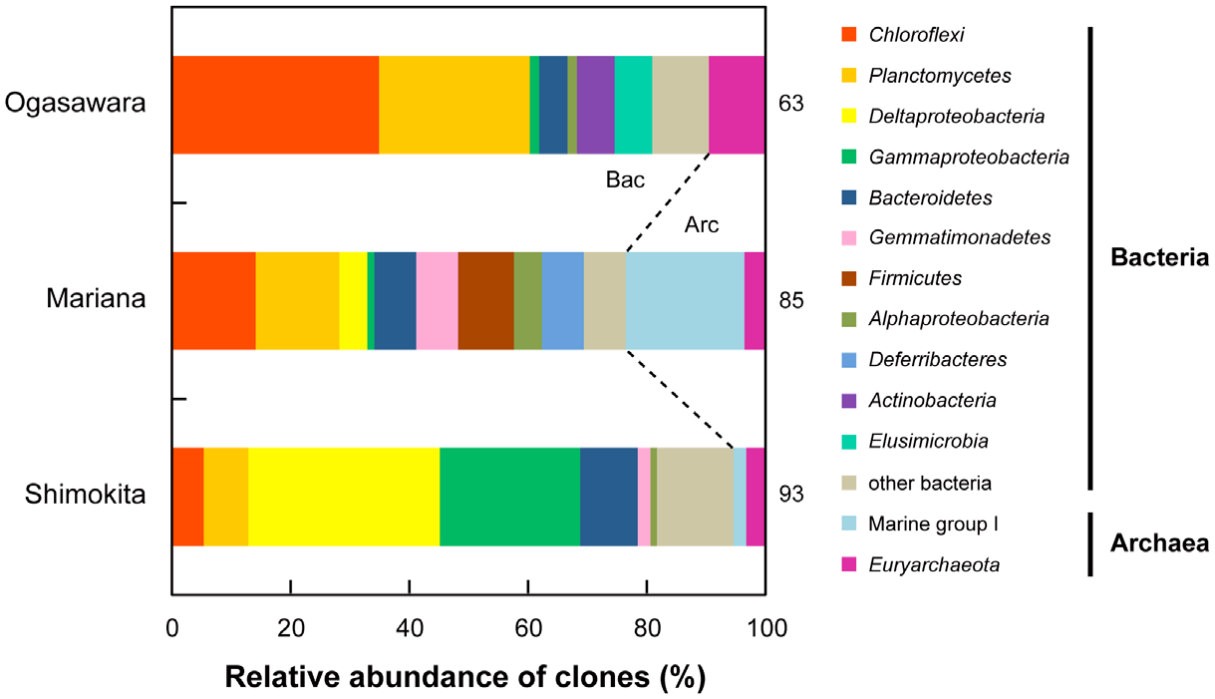
Prokaryotic community structures based on the bacterial and archaeal 16S rRNA gene clone sequences detected from the deep-sea shallow subseafloor sediments from the Ogasawara, Mariana, and Shimokita locations. The numbers on the right of each row show the numbers of the sequenced clones in each library. The “Others” category represents the bacterial taxa that compose less than 5% of the total clone numbers.

The phylotype compositions of the prokaryotic communities in the deep-sea shallow subseafloor sediment samples that hosted relatively abundant virus populations were assessed by 16S rRNA gene clone analysis. Most of the 16S rRNA gene phylotypes recovered from the sediments were derived from the previously uncultivated prokaryotes but were related to environmental sequences that have frequently been identified in deep-sea surface and subseafloor sedimentary habitats. In addition, the proportions of the phylum-level compositional groups in the 16S rRNA gene clone libraries (Fig. 2) were different between the three sedimentary habitats, but a considerable portion of their constituent phylotypes was identified commonly among the deep-sea shallow subseafloor sediments. The predominant phylogroups in the SH sediment were *Deltaproteobacteria* (32%) and *Gammaproteobacteria* (24%) (Fig. 2). In contrast, phylotypes affiliated with *Chloroflexi* (35%) and *Planctomycetes* (25%) dominated the phylotype composition of the OG sediment. In the MA sediment, the phylotypes of the marine group I archaea represented the most predominant prokaryotic components (20%) (Fig. 2).

### Compositions of the Viromes

**Figure 3:**
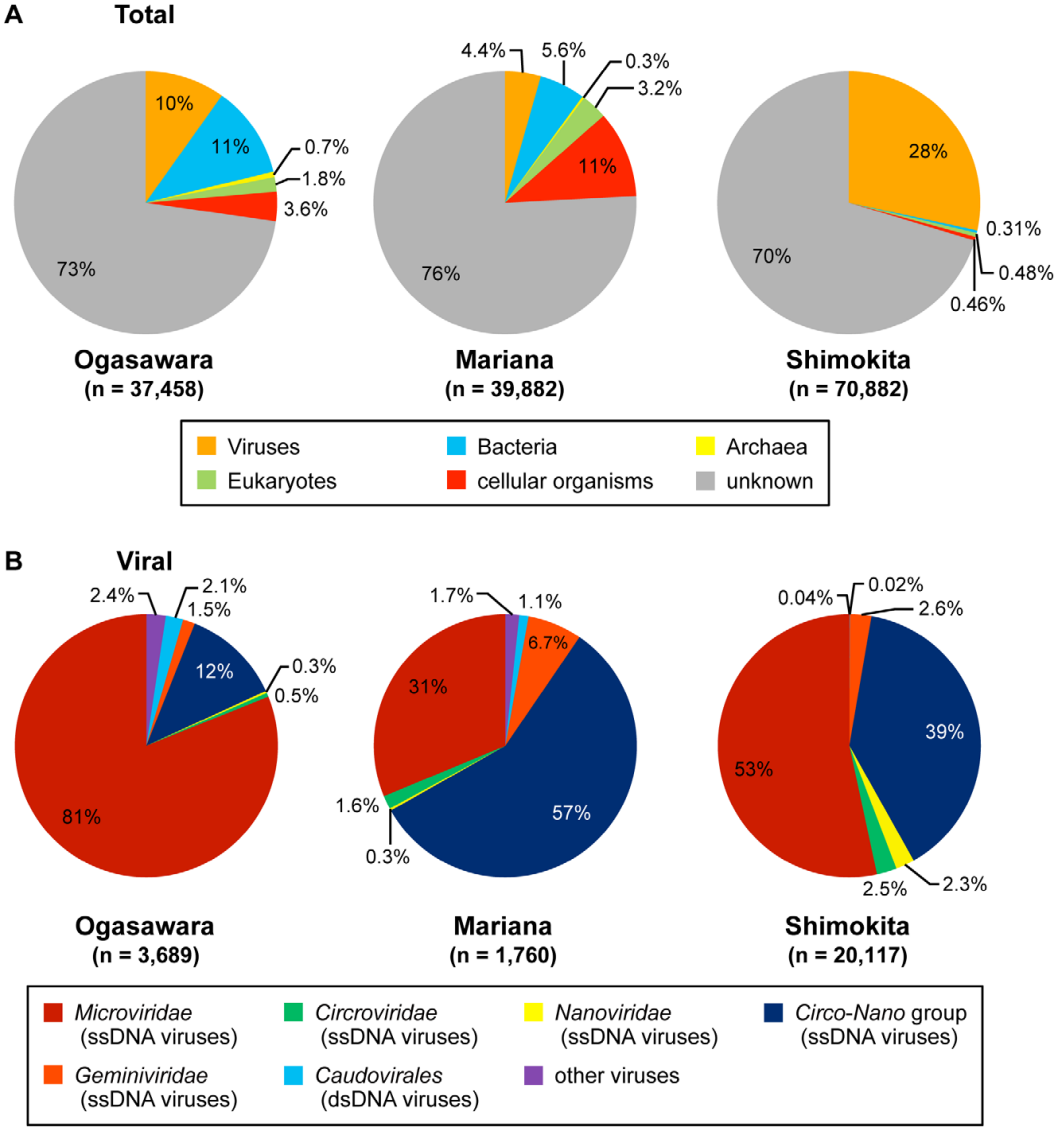
Taxonomic composition of the sequence reads in the virome libraries from the deep-sea shallow subseafloor sediments. (A) Relative abundance of the sequence reads classified by the taxonomic grouping based on BLASTx similarity search (E-value <10^−3^). Sequences with no hits or hits with E-values >10^−3^ were regarded as unidentified reads (“unknown” category in the pie graphs). (B) Relative abundance of the sequence reads most similar to previously identified viral families (E-value <10^−3^ in BLASTx). The “Other” category represents the unclassified viruses and viral families constituting less than 1% of the total viral reads.

A total of 37,458, 39,882, and 70,882 sequence reads were obtained from the pyrosequencing libraries of the three surface sedimentary viromes in the OG, MA, and SH sediments, respectively (Table 1). Only 24−30% of the sequence reads in the libraries exhibited significant similarity (E-value <10^−3^ in BLASTx) to the sequences deposited in the GenBank nr protein database (Fig. 3A), and these reads were further classified into viral, prokaryotic, or eukaryotic sequences based on the top 10% of the significant hits (Fig. 3A). In the SH pyrosequencing library, a relatively higher proportion (28%) of reads were assigned as of potentially viral origins, and either 0.3% or 0.5% of the reads were categorized as being of potential bacterial or eukaryotic origins. The potentially viral origin of reads was found in 10% and 4% of the OG and MA pyrosequencing libraries, respectively, while 11% and 6% of the OG and MA pyrosequencing libraries, respectively, were likely derived from a bacterial origin. However, as reported in other environmental virome studies (e.g., [19], [21], [35], [63]), the similarity analysis revealed that most of the sequences in all of the pyrosequencing libraries were unclassified (Fig. 3A).

**Table 1:** Overview of the sequence reads and the assembled contigs of the virome pyrosequencing libraries analyzed in this study.

	Read data				Contig data*	
Sampling site	Number of reads	Total length (Mb)	Average length (bp)	GC content (%)	Number of contigs	Total length (Mb)
Ogasawara (OG)	37,458	10.8	289	45.1	17,734	5.7
Mariana (MA)	39,882	10.1	253	52.1	15,602	4.5
Shimokita (SH)	70,882	21.2	212	45.0	24,799	8.7

* The data contained unassembled sequences (singletons).

The potentially virus-derived sequences were further classified into sequences tentatively associated with the family-level taxa of viruses (Fig. 3B). Most of the viral reads in all three libraries were found to be genetic components from ssDNA viral families, including *Microviridae*, *Circoviridae*, *Nanoviridae*, and *Geminiviridae*. These tentative ssDNA viral sequences together occupied 95−99% of the total viral reads in each library (Fig. 3B). The ssDNA viral sequences from the OG sediment were related to the genetic components from *Microviridae* phages (81% of the total viral reads), whereas 59% of the total viral reads from the MA sediment library were likely derived from the *Circoviridae*−*Nanoviridae* viral group, which is known to infect eukaryotes [34]. In the SH sediment library, the sequences associated with both viral groups (*Microviridae* and *Circoviridae*−*Nanoviridae* groups) were predominant (53% and 44% of the total viral reads, respectively). In contrast, the possible viral reads related to dsDNA viruses, including the order *Caudovirales*, known as “tailed bacteriophages”, were detected as very minor populations (0.03−3.2% of the total viral reads) in the three libraries.

### Profile of Functional Genes from the Viromes

All of the constituent sequence reads of the genes predicted from the deep-sea shallow subseafloor sedimentary viromes were subjected to functional assignments based on the SEED-subsystems (Fig. S1). Most of the functionally assigned sequences among all libraries belonged to the viral protein category (37−98% of the total reads assigned). Only a small fraction of the sequences from the OG and MA sediment libraries were classified into various functional categories, including microbial metabolism (Fig. S1). The sequences assigned as the viral protein category were further subgrouped into several subcategories (Table 2). A majority (76−94%) of the viral genes from each library were classified into three ssDNA viral protein categories: replication proteins, major capsid proteins, and minor capsid proteins (Table 2).

**Table 2:** Relative abundance of the constituent sequence reads of the genes predicted from the deep-sea sediment viromes assigned to viral protein categories.

Description	Ogasawara	Mariana	Shimokita
Replication protein	25.8%	47.5%	49.1%
Major capsid protein	32.1%	18.1%	32.2%
Minor capsid protein	21.0%	10.5%	12.5%
Capsid protein	0.8%	11.1%	0.4%
Nonstructural protein	4.2%	3.6%	4.0%
Endonuclease	0.7%	0.6%	0%
DNA methylase	0.7%	0.2%	0%
Crossover junction endodeoxyribonuclease	0.8%	0%	0%

The viral protein categories shared more than 0.5% of the total viral reads are presented.

### Diversity of ssDNA Viral Sequences in the Viromes

The genetic diversity of the ssDNA viral sequences obtained from three libraries was examined with the MetaVir tool [56], enabling the detection of the diversity of representative viral marker genes (Table S1). As a result, only three viral makers were identified, and these markers are summarized in Table 3: conserved major capsid protein (VP1) of *Microviridae* phages [35], a putative replication initiation protein (Rep) of the eukaryotic infectious *Circoviridae*−*Nanoviridae*−*Geminiviridae* group [34], and a terminase large subunit (TerL) [64] of the dsDNA viruses affiliated with *Caudovirales*. A high genotypic diversity of these two ssDNA viral sequence groups was found (833 genotypes for VP1 and 2,551 genotypes for Rep).

**Table 3:** Distribution of the viral genetic marker-related sequences from the virome assemblies obtained through the MetaVir workflow [56].

Viromes | Markers	G20	GP23	MCP	PolB	PsbA	RdRP	Rep	T7gp17	TerL	VP1
OG	0	0	0	0	0	0	57 (119)	0	2 (5)	255 (1,179)
MA	0	0	0	0	0	0	44 (124)	0	0	127 (212)
SH	0	0	0	0	0	0	732 (2,277)	0	1 (1)	2,169 (4,497)

The data show the abundance of the assembled contigs (>150 bp) related to the viral marker genes. The contigs were assembled from the virome read sequences (E-value <10^−3^) that were homologous to previously known viral PFAM references. The numbers in parentheses indicate the abundance of the constituent reads of the sequences.

Based on the MetaVir data, we selected two ssDNA viral markers (the VP1 and Rep genes) to construct the phylogenetic trees to determine the phylogenetic relationship between the potential deep-sea shallow subseafloor sedimentary ssDNA viruses and previously identified viruses, including environmental sequences. From the virome CDSs identified by multiple informative programs (e.g., MetaGeneMark [49]) for gene finding from metagenomic sequences, we screened 100, 35, and 686 CDSs encoding partial or complete viral VP1 genes and 85, 57, and 784 CDSs encoding partial or complete putative viral Rep genes from the OG, MA, and SH virome libraries, respectively. Then, three conserved domains in the VP1 and Rep genes were explored on the Pfam website: the major capsid protein F domain (PF02305, Phage_F) in VP1 and the putative viral replication protein domain (PF02407, Rep Viral) and Geminivirus Rep protein catalytic domain (PF00799, Gemini_AL1) in Rep. Consequently, we obtained 11 (from the OG library), 7 (from the MA library), and 71 (from the SH library) CDSs that harbored at least one complete or nearly complete conserved domain (7, 2, and 36 sequences for the major capsid protein F domain; 4, 5, and 72 sequences for the putative viral replication protein domain; and 0, 0, and 17 sequences for the Geminivirus Rep protein catalytic domain).

**Figure 4:**
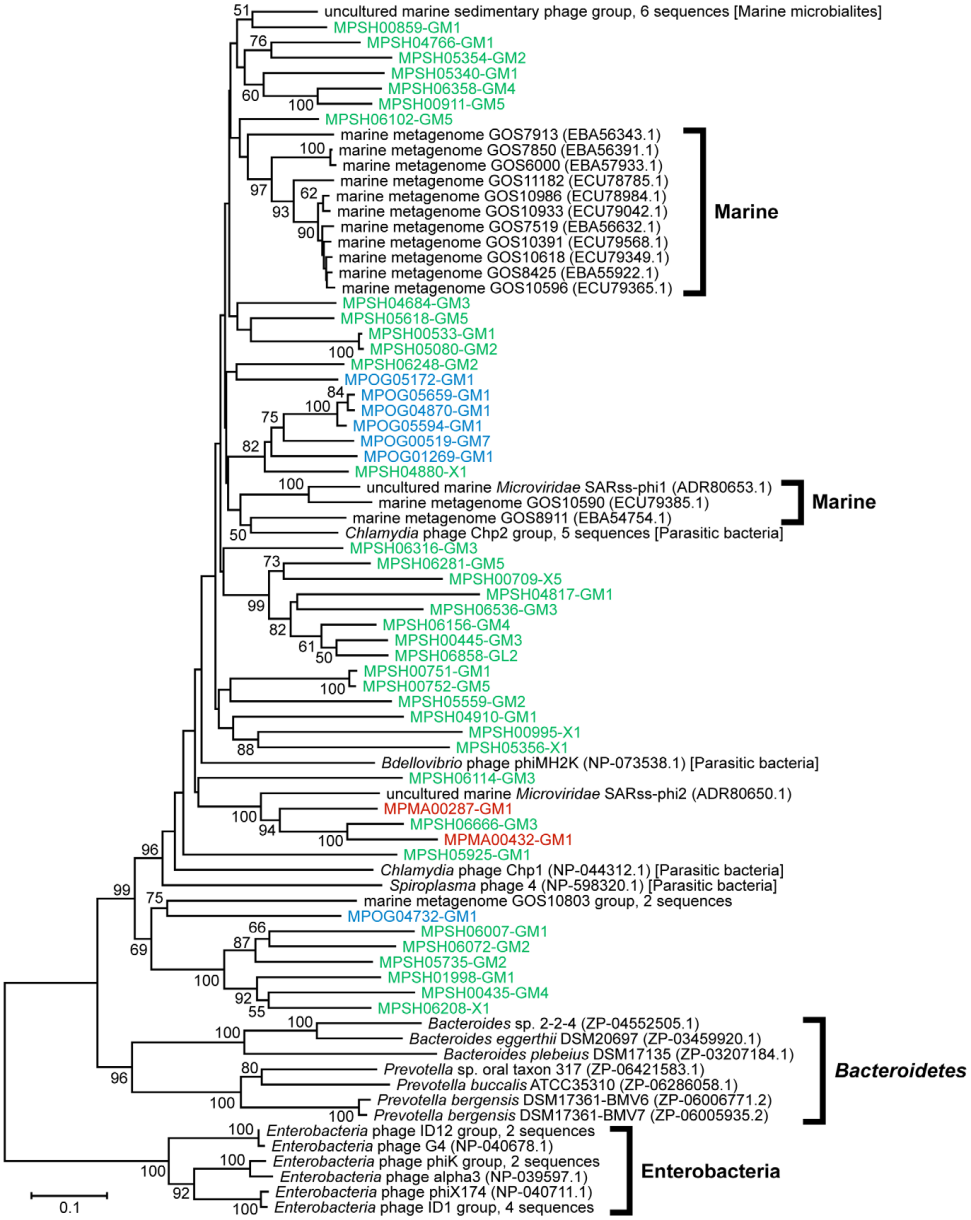
The neighbor-joining phylogenetic tree of the 136 amino acid sequences from the *Microviridae* Phage_F domain (pfam02305). The virome sequences from the Ogasawara (OG), Mariana (MA), and Shimokita (SH) libraries are highlighted in blue, red, and green, respectively. The numbers in parentheses indicate the DDBJ/EMBL/GenBank accession numbers for the sequences. Only bootstrap values of >50% are indicated at the nodes of the tree. The uncultured marine sedimentary phage group corresponds with six environmental clones: A1 (accession no. ABS86616.1), A2 (accession no. ABS86617.1), A3 (accession no. ABS86618.1), A4 (accession no. ABS86619.1), B3 (accession no. ABS86620.1), and B4 (accession no. ABS86621.1). The *Chlamydia* phage Chp2 group corresponds with five isolates: Chp2 (accession no. NP_054647.1), Chp3 (accession no. YP_022479.1), Chp4 (accession no. YP_338238.1), CPAR39 (accession no. NP_063895.1), and phiCPG1 (accession no. NP_510872.1). The marine metagenome GOS10803 group corresponds with two environmental clones: GOS10803 (accession no. ECU79166.1) and GOS11146 (accession no. ECU78830.1). The *Enterobacteria* phage phiK group corresponds with two isolates: phiK (accession no. Q38041.1) and St-1 (accession no. YP_002985212.1). The *Enterobacteria* phage ID12 group corresponds with two isolates: ID12 (accession no. AAZ49343.1) and WA6 (accession no. AAZ49332.1). The *Enterobacteria* phage ID1 group corresponds with four isolates: ID1 (accession no. AAZ49068.1), NC11 (accession no. AAZ49145.1), S13 (accession no. AAG29961.1), and WA10 (accession no. AAZ49222.1).

**Figure 5:**
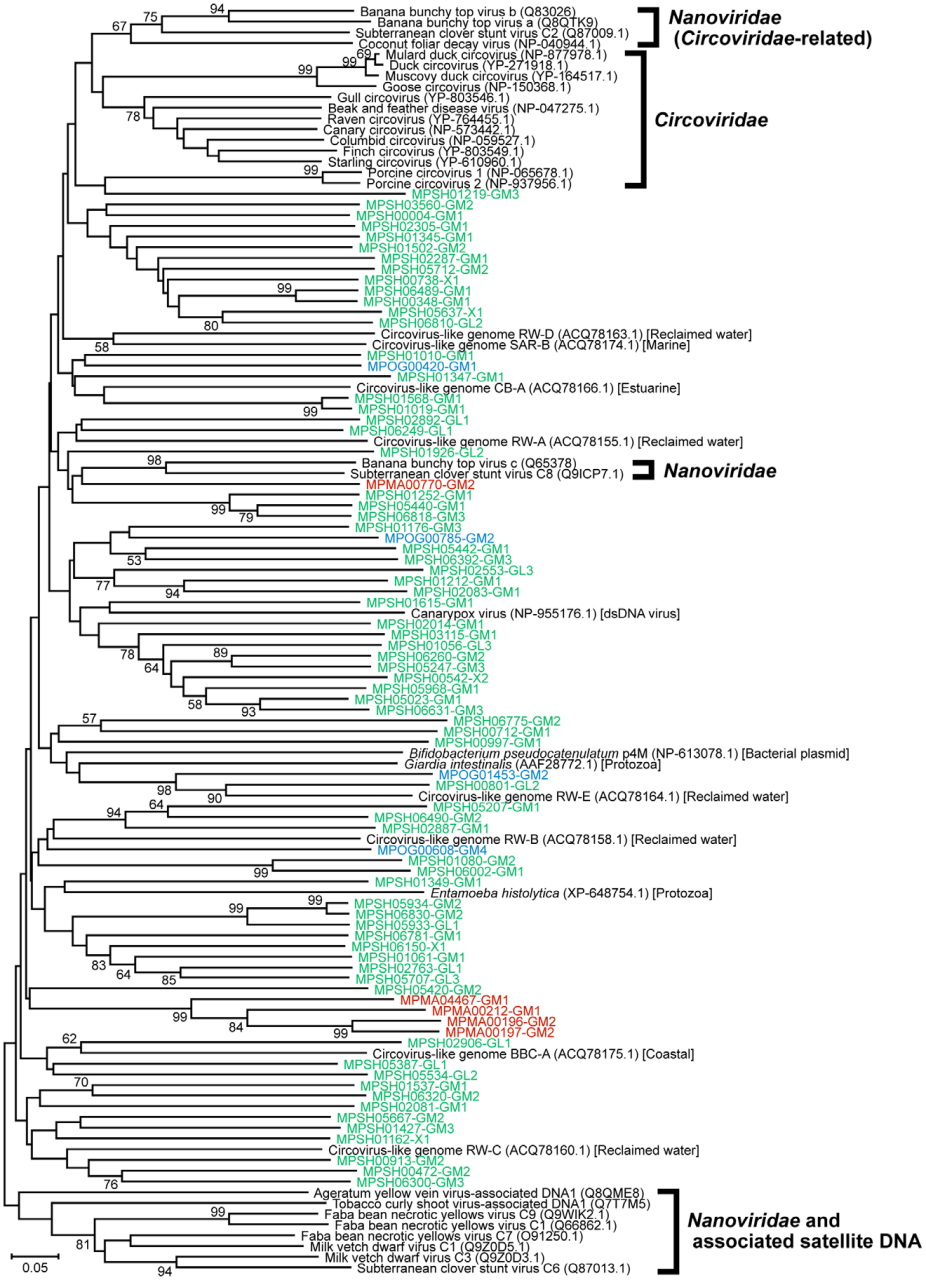
The neighbor-joining phylogenetic tree of the 101 amino acid sequences from the Rep_Viral domain (pfam02407). The virome sequences from the Ogasawara (OG), Mariana (MA), and Shimokita (SH) libraries are highlighted in blue, red, and green, respectively. The numbers in parentheses indicate the DDBJ/EMBL/GenBank accession numbers for the sequences. Only bootstrap values of >50% are indicated at the nodes of the tree.

**Figure 6:**
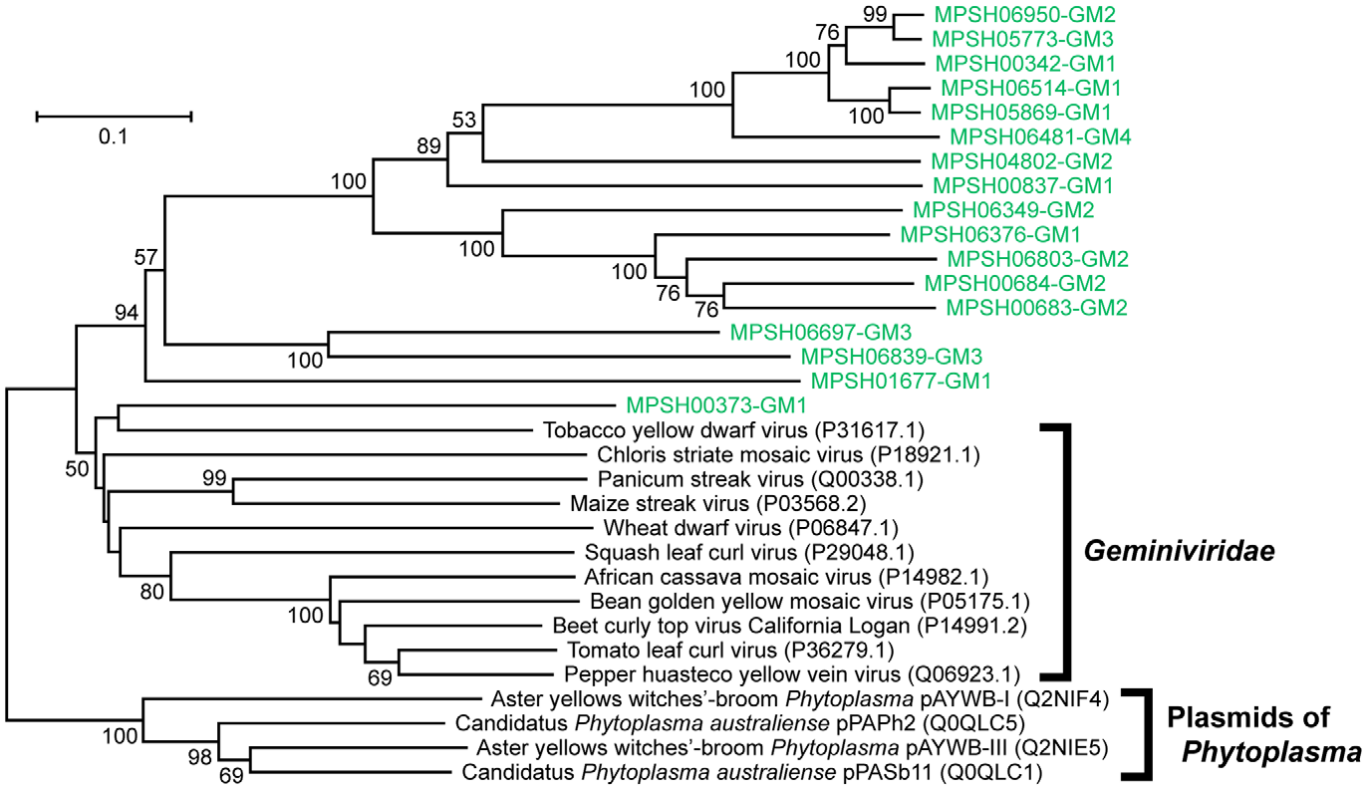
The neighbor-joining phylogenetic tree of the 119 amino acid sequences from the Gemini_AL3 domain (pfam00799). The virome sequences from the Shimokita (SH) library are highlighted in green. The numbers in parentheses indicate the DDBJ/EMBL/GenBank accession numbers for the sequences. Only bootstrap values of >50% are indicated at the nodes of the tree.

We constructed a phylogenetic tree for each viral marker gene domain (Fig. 4, 5, 6). The phylogenetic tree of the *Microviridae*-capsid protein F domain revealed that the sequences obtained in this study were more closely related to sequences from an intracellular parasitic bacteria-infectious phage group (*Chlamydia*, *Bdellovibrio*, and *Spiroplasma* phages) or environmental sequence groups detected in oceanic waters and marine sedimentary microbialites compared with those from the *Enterobacteria* and *Bacteroidetes* phage groups (Fig. 4). However, our benthic virome sequences did not fall within any groups composed of known *Microviridae* sequences (Fig. 4). The phylogenetic tree of the viral_Rep domain group indicated that the sequences obtained from the deep-sea shallow subseafloor sediments were very diverse and that most were distinct from previously characterized ssDNA viral groups (Fig. 5). In addition, the phylogenetic analysis of the Gemini_AL1 domain revealed that the sequences identified in this study were moderately related to the known members of the *Geminiviridae* family and that all the virome sequences, with the exception of MPSH00373, formed a novel phylogenetic cluster (Fig. 6).

We also employed an alternative approach to examine the ssDNA viral diversity by using the automatic tree construction tool in MetaVir [56]. In contrast to the above-mentioned phylogenetic analysis, which was performed with sequences that were as long as possible, this tree construction tool has been developed to analyze as many metagenomic sequences as possible in phylogenetic trees with reference sequences for each genetic marker (for details, see Materials and Methods S2 in the supplemental material). Representative reliable phylogenetic trees of the VP1 and Rep sequences, including relatively abundant genotypes obtained in this study, are shown in Fig. S2 and Fig. S3, respectively. The phylogenetic trees also indicated that the VP1 and Rep sequences from the deep-sea shallow subseafloor sediments were phylogenetically distinct from those of any previously known *Microviridae* phages and eukaryotic infectious ssDNA viruses. The results also supported the phylogenetic topology and diversity found in the trees constructed from longer sequences (Fig. 4, 5, 6).

### Comparison of Various Viromes

**Figure 7:**
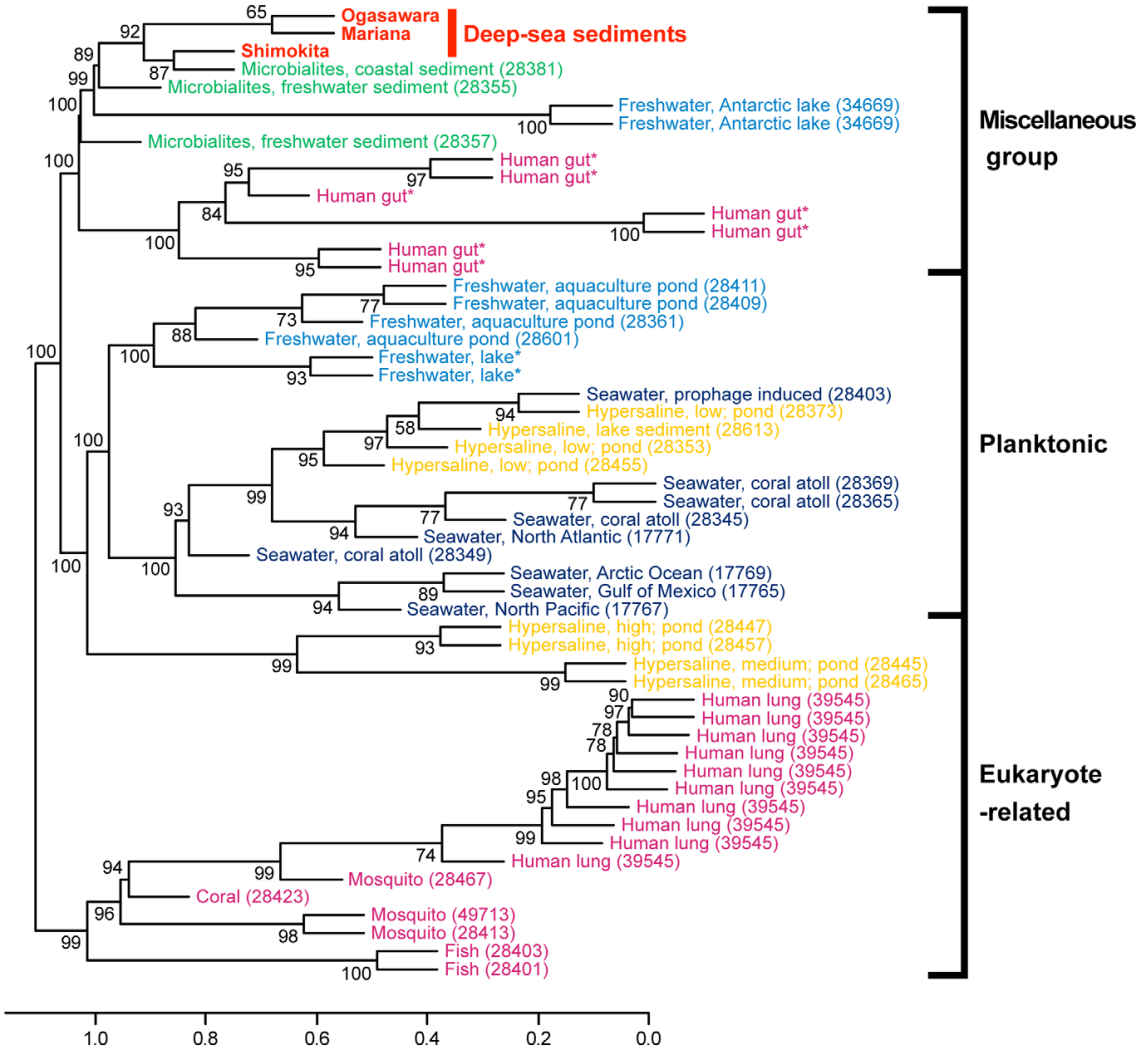
Dendrogram of three deep-sea sedimentary viromes and other environmental viromes based on the BLAST sequence similarity. Only bootstrap values of >50% are indicated at the nodes of the dendrogram. A subgroup that included the deep-sea shallow subseafloor sedimentary viromes and with a height of 1.04 and high bootstrap support (>95%) was found in the dendrogram. The deep-sea shallow subseafloor sedimentary viromes are highlighted in red, and the viromes from other environments are colored according to their ecosystem. The numbers in parentheses indicate the ID numbers of the genome project in NCBI. *public virome project available in the MetaVir server.

Based on a cross-tBLASTx search for sequence similarities among any of the available virome libraries, we compared the three deep-sea shallow subseafloor sedimentary viromes to viromes from other environments (Fig. 7). The cluster analysis revealed that the presently known viromes could be classified into three representative groups: planktonic viromes (e.g., viromes in seawater and freshwater), eukaryote-associated viromes (e.g., fish, mosquito, and human lung viromes), and another miscellaneous group (deep-sea sediment, microbialite, Antarctic ultra-oligotrophic freshwater, and human gut viromes) (Fig. 7). The classification was supported with high bootstrap values (>95%) at a cutoff value of 1.04 on the dendrogram. In the miscellaneous virome group, the three deep-sea shallow subseafloor sedimentary viromes were closely related to each other and could generate a specific subgroup with the virome from the coastal sedimentary microbialite (Fig. 7).

## Discussion

Most of the potentially virus-originating sequences from the deep-sea shallow subseafloor sediments were similar to sequences from ssDNA viruses, such as the families of *Microviridae*, *Circoviridae*, *Nanoviridae*, and *Geminiviridae* (Fig. 3B). These ssDNA viruses have been isolated only from terrestrial environments; however, recently, both isolation and metagenomic studies have revealed the existence of ssDNA viruses in marine environments. To date, seven ssDNA viruses infecting marine diatoms (the new genus *Bacilladnavirus*) [65]–[68] have been isolated, and Holmfeldt et al. [69] reported the first description of *Microviridae* phages that infect marine *Bacteroidetes* (*Cellulophaga baltica*). Furthermore, diverse ssDNA viral-related sequences have also been recovered in metagenomic investigations of marine environments, such as oceanic waters [19], [38], [70], coastal microbialites [35], coral [36], and marine protist cells [71].

Of the ssDNA viral families phylogenetically associated with the sequences from the deep-sea shallow subseafloor sediments, only *Microviridae* is a bacteriophage family, whereas the other families are known as eukaryotic viral families; *Circoviridae* infects animals, and *Nanoviridae* and *Geminiviridae* infect plants. The phylogenetic analyses of the virome genes revealed that the viromes in the deep-sea shallow subseafloor sediments harbored diverse phage VP1- and viral Rep-related sequences (Table 3) that were genetically distinct from the previously known *Microviridae* phages and eukaryotic infectious ssDNA groups and their homologs identified by metagenomic characterizations of other environments (e.g., oceanic and fresh waters) (Fig. 4, 5, 6; see also Figs. S2 and S3 in the supplemental material). In eukaryotic ssDNA viruses, the Rep protein family is known to include non-viral replication-associated proteins from bacterial plasmids (*Bifidobacterium pseudocatenulatum* pM4 and *Phytoplasma* sp.) and protozoan genomes (*Giardia intestinalis* and *Entamoeba histolytica*) [72]. In particular, the genetic diversity of the Rep genes obtained from the deep-sea sedimentary viromes suggests that the potential ssDNA viruses harboring the Rep genes would have much greater diversity in host selection than presently expected [73], [74]. Thus, the genetically diverse ssDNA virus candidates in the deep-sea shallow subseafloor sediments may infect not only eukaryotic but also prokaryotic hosts, although it is still unclear whether such potential ssDNA viruses actively interact with the host eukaryotic and prokaryotic populations in the *in situ* sedimentary habitats or other ocean environments.

The proportion of ssDNA viral sequences among all the possible viral sequences is significantly higher (95−99%) in the deep-sea sedimentary viromes (Fig. 3B) than in other previously described ocean planktonic viromes [19]. However, it should also be noted that the predominance of ssDNA viral sequences in the deep-sea sedimentary viromes may be biased by the method (MDA method) used to construct the virome library in this study. The MDA method has been adopted in several metagenomic studies of planktonic viromes in seawater samples of the Arctic Ocean, Gulf of Mexico, British Columbia, and Sargasso Sea, and a lower proportion (0.7 to 25.0%) of ssDNA viral sequences among the whole viral sequences has been demonstrated [19]. Thus, although methodological biases cannot be completely excluded, the comparison of the results of the ocean planktonic and benthic viromes suggests the potential predominance of the ssDNA viral components in the viral populations of the (hado)pelagic sedimentary habitats.

Although a high abundance of ssDNA viral sequences was commonly noted in the viromes of the (hado)pelagic sedimentary habitats, many differences also became evident upon the detailed comparison of the three deep-sea sedimentary viromes (Fig. 3B). For example, the MA virome library was dominated by sequences related to ssDNA viruses of the eukaryotic infectious *Circoviridae*−*Nanoviridae* group, while the OG virome library was dominated by *Microviridae*-related sequences (Fig. 3B). We expected that the difference in viral genotype compositions was most likely associated with the different host community compositions, specifically, the compositional differences between prokaryotic communities as the predominant microbial populations in the deep-sea sedimentary habitats. The 16S rRNA gene phylotype analysis revealed a difference in the phylum-level composition of the prokaryotic phylotypes but a considerably similar pattern of the emerging constituent phylotypes in the three deep-sea shallow subseafloor sediments (Fig. 2), so that we could not find how the viral genotype compositions are coupled with the potential host microbial (prokaryotic and eukaryotic) community compositions in the deep-sea shallow subseafloor sediments.

In contrast, a relatively high abundance of sequences potentially originating from bacteria was indicated in the virome libraries of the OG and MA sediments (11% and 6%, respectively), and each of the three viromes represented a unique composition of viral and non-viral sequence origins (Fig. 3A). In previous metagenomic virome studies, the sequences identified as of non-viral origin, such as prokaryotic and eukaryotic sequences, were interpreted to be the result of a potential misclassification of viral sequences as host (prokaryote and eukaryote) genomic components [19]–[21], [27], [28], [75]. However, in this study, the non-viral sequences found in the viromes may be derived from the potential contamination of the extracellular DNA by the indigenous prokaryotic and eukaryotic populations during the viral purification processes. To purify viral particles from the sediment samples, we used the CsCl density centrifugation method but did not perform DNase digestion of the extracellular free DNA fragments in the purified viral fractions. The functional profiling of the virome sequences (Fig. S1) revealed that the genes related to virus-mediated gene transfer, such as those encoding integrases and transposases and belonging to the category of prophages and transposable elements, were rarely observed (1.4% and 0.2% in the OG and MA virome libraries, respectively). Because the viral abundance was significantly lower in the OG and MA sediments (6.6×10^7^ and 1.2×10^7^−5.3×10^7^ viruses/cm^3^, respectively) than the SH sediment (7.6×10^10^ viruses/cm^3^) (Table S2), the influence of contaminated extracellular DNA would be greater in the OG and MA virome libraries than the SH virome library. Therefore, the bacterial sequences identified in this study may be due to contamination by extracellular DNA from cellular organisms.

The deep-sea shallow subseafloor sedimentary viromes were compared with previously characterized viromes of other environments by pairwise sequence similarities using the MetaVir workflow (Fig. 7). Because the analysis addresses not only sequences of known function but also sequences of unknown function, which constitute most of the virome sequences in public databases, the MetaVir analysis can provide a comparison between viromes with a broader spectrum of genetic information. The cluster analysis revealed that all of the deep-sea shallow subseafloor sedimentary viromes and coastal microbialite virome form a novel group of viromes that are clearly differentiated from the viromes of other environments, particularly the aquatic (marine and freshwater) viromes (Fig. 7). The distinct characteristics of the deep-sea shallow subseafloor sedimentary viromes in the statistical analysis are consistent with the domination of the novel viral genotype compositions by sequences from ssDNA viruses (Fig. 3B).

Although many differences in the virome compositions (e.g., the detailed viral genotype composition [Fig. 3B]) and environmental conditions (e.g., geographical location, geological and oceanographic settings, physical and chemical environments, and potential prokaryotic community structures [Fig. 2]) were identified, the deep-sea shallow subseafloor sedimentary viromes were statistically related to each other (Fig. 7). It is interesting that the viromes in the extant microbialite habitats have a significant relationship with the deep-sea sedimentary viromes (Fig. 7). The microbialites are types of complex sedimentary mineral and microbial structures that grow with photosynthetic primary production and the associated heterotrophic populations and are controlled microbially by mineral deposition in coastal and freshwater environments [35]. The microbialite virome in a shallow coastal area has been characterized by the high abundance of previously known viral sequences from ssDNA viruses and several marine cyanophages [35]. The deposition rates and properties and the indigenous microbial processes appear to differ considerably between the (hado) pelagic sediments and the microbialites, whereas both of the aquatic sedimentary habitats may have similar environmental and microbiological interactions in the development of the *in situ* viral community.

Here, we described the characteristics of viromes in deep-sea sediments. The virome investigations revealed that the (hado)pelagic sediments harbored novel viromes, including previously unidentified ssDNA viruses distinct from the viral genotypes previously identified in ocean environments, although the relative abundance of these ssDNA viral assemblages were likely biased during the construction of the metagenomic library. Still now, prospective trials of less biased methods to prepare the virome library con­tinue to be developed [76]–[78], including new co-purification methods allowing simultaneous access to dsDNA, ssDNA, and RNA viruses from the same sample [79]. Therefore, further advanced investigations of community metagenomes of multiple DNA and RNA viral families are required to obtain a more comprehensive and reliable overview of the viral community in the deep-sea sedimentary environment. Moreover, in-depth analyses of the viral and host microbial community metagenome datasets in the (hado)pelagic sedimentary zones would provide a better understanding of the host-virus systems in the deep-sea sediments.

## Supporting Information

Figure S1 **Profiles of the function categories for the genes predicted from three deep-sea shallow subseafloor sedimentary viromes.** The relative abundance of the constituent sequence reads of the virome genes assigned to SEED subsystems [51] with significance (E-value <10^−3^ in BLASTp) is shown.
(TIF)

Figure S2 **Maximum-likelihood tree of the 58 amino acid sequences of the major capsid protein (VP1 marker for** ***Microviridae*** **) from the contigs in the virome libraries, as represented by a tree gallery (the 50 ‘best’ trees) with the MetaVir workflow** [**56**] **.** The virome sequences from the Ogasawara (OG), Mariana (MA), and Shimokita (SH) libraries are highlighted in blue, red, and green, respectively. The numbers in parentheses indicate the DDBJ/EMBL/GenBank accession numbers for the sequences. Only bootstrap values of >50% are indicated at the nodes of the tree.
(TIF)

Figure S3 **The neighbor-joining phylogenetic tree of the 52 amino acid sequences of the replication protein (Rep marker for the** ***Circoviridae*** **−** ***Nanoviridae*** **−** ***Geminiviridae*** **group) from the contigs in the virome libraries as represented by a tree gallery (the 50 ‘best’ trees) with the MetaVir workflow** [**56**] **.** The virome sequences from the Ogasawara (OG), Mariana (MA), and Shimokita (SH) libraries are highlighted in blue, red, and green, respectively. The numbers in parentheses indicate the DDBJ/EMBL/GenBank accession numbers for the sequences. Only bootstrap values of >50% are indicated at the nodes of the tree.
(TIF)

Table S1 **Representative viral genetic markers used in the MetaVir workflow** [**56**] **.**
(DOC)

Table S2 **Viral abundance in the (hado)pelagic surface sediments (down to 30 cmbsf) from the Ogasawara, Mariana, and Shimokita locations.**
(DOC)

Materials and Methods S1 **Prokaryotic 16S rRNA gene clone library analysis.**
(PDF)

Materials and Methods S2 **Online phylogenetic analysis of the ssDNA viral sequences with MetaVir.**
(PDF)

## References

[1] Fuhrman JA (1999) Marine viruses and their biogeochemical and ecological effects. Nature 399: 541–548. 10376593 10.1038/21119

[2] Wommack KE, Colwell RR (2000) Virioplankton: Viruses in aquatic ecosystems. Microbiol Mol Biol Rev 64: 69–114. 10704475 10.1128/mmbr.64.1.69-114.2000 PMC98987

[3] Bouvier T, del Giorgio PA (2007) Key role of selective viral-induced mortality in determining marine bacterial community composition. Environ Microbiol 9: 287–297. 17222128 10.1111/j.1462-2920.2006.01137.x

[4] Faruque SM, Naser IB, Islam MJ, Faruque AS, Ghosh AN, et al (2005) Seasonal epidemics of cholera inversely correlate with the prevalence of environmental cholera phages. Proc Natl Acad Sci U S A 102: 1702–1707. 15653771 10.1073/pnas.0408992102 PMC547864

[5] Sandaa RA, Larsen A (2006) Seasonal variations in virus-host populations in Norwegian coastal waters: Focusing on the cyanophage community infecting marine *Synechococcus* spp. Appl Environ Microbiol 72: 4610–4618. 16820451 10.1128/AEM.00168-06 PMC1489308

[6] Suttle CA (2007) Marine viruses−major players in the global ecosystem. Nat Rev Microbiol 5: 801–812. 17853907 10.1038/nrmicro1750

[7] Yau S, Lauro FM, DeMaere MZ, Brown MV, Thomas T, et al (2011) Virophage control of antarctic algal host-virus dynamics. Proc Natl Acad Sci U S A 108: 6163–6168. 21444812 10.1073/pnas.1018221108 PMC3076838

[8] Yoshida M, Yoshida T, Kashima A, Takashima Y, Hosoda N, et al (2008) Ecological dynamics of the toxic bloom-forming cyanobacterium *Microcystis aeruginosa* and its cyanophages in freshwater. Appl Environ Microbiol 74: 3269–3273. 18344338 10.1128/AEM.02240-07 PMC2394914

[9] Yoshida M, Yoshida T, Yoshida-Takashima Y, Kashima A, Hiroishi S (2010) Real-time PCR detection of host-mediated cyanophage gene transcripts during infection of a natural *Microcystis aeruginosa* population. Microbes Environ 25: 211–215. 21576874 10.1264/jsme2.me10117

[10] Fuhrman JA, Noble RT (1995) Viruses and protists cause similar bacterial mortality in coastal seawater. Limnol Oceanogr 40: 1236–1242.

[11] Nagata T (2000) Production mechanisms of dissolved organic matter. In: Kirchman DL, editor. Microbial ecology of the oceans. New York: Wiley-Liss. 121–152.

[12] Suttle CA (2005) Viruses in the sea. Nature 437: 356–361. 16163346 10.1038/nature04160

[13] Sullivan MB, Lindell D, Lee JA, Thompson LR, Bielawski JP, et al (2006) Prevalence and evolution of core photosystem II genes in marine cyanobacterial viruses and their hosts. PLoS Biol 4: e234. 16802857 10.1371/journal.pbio.0040234 PMC1484495

[14] Weinbauer MG (2004) Ecology of prokaryotic viruses. FEMS Microbiol Rev 28: 127–181. 15109783 10.1016/j.femsre.2003.08.001

[15] Corinaldesi C, Crevatin E, Del Negro P, Marini M, Russo A, et al (2003) Large-scale spatial distribution of virioplankton in the Adriatic Sea: Testing the trophic state control hypothesis. Appl Environ Microbiol 69: 2664–2673. 12732535 10.1128/AEM.69.5.2664-2673.2003 PMC154510

[16] Gage JD, Tyler PA (1991) Deep-sea biology: A natural history of organisms at the deep-sea floor. Cambridge, UK: Cambridge University Press. 504 p.

[17] Danovaro R, Corinaldesi C, Filippini M, Fischer UR, Gessner MO, et al (2008) Viriobenthos in freshwater and marine sediments: A review. Freshwater Biol 53: 1186–1213.

[18] Danovaro R, Dell’Anno A, Corinaldesi C, Magagnini M, Noble R, et al (2008) Major viral impact on the functioning of benthic deep-sea ecosystems. Nature 454: 1084–1087. 18756250 10.1038/nature07268

[19] Angly FE, Felts B, Breitbart M, Salamon P, Edwards RA, et al (2006) The marine viromes of four oceanic regions. PLoS Biol 4: 2121–2131. 10.1371/journal.pbio.0040368 PMC1634881 17090214

[20] Dinsdale EA, Edwards RA, Hall D, Angly F, Breitbart M, et al (2008) Functional Metagenomic Profiling of Nine Biomes. Nature 452: 344–347. 18337718 10.1038/nature06810

[21] Lopez-Bueno A, Tamames J, Velazquez D, Moya A, Quesada A, et al (2009) High diversity of the viral community from an Antarctic lake. Science 326: 858–861. 19892985 10.1126/science.1179287

[22] Ng TFF, Willner DL, Lim YW, Schmieder R, Chau B, et al (2011) Broad surveys of DNA viral diversity obtained through viral metagenomics of mosquitoes. PLoS ONE 6: e20579. 21674005 10.1371/journal.pone.0020579 PMC3108952

[23] Rodriguez-Brito B, Li L, Wegley L, Furlan M, Angly F, et al (2010) Viral and microbial community dynamics in four aquatic environments. ISME J 4: 739–751. 20147985 10.1038/ismej.2010.1

[24] Vega Thurber RL, Barott KL, Hall D, Liu H, Rodriguez-Mueller B, et al (2008) Metagenomic analysis indicates that stressors induce production of herpes-like viruses in the coral *Porites compressa* . Proc Natl Acad Sci U S A 105: 18413–18418. 19017800 10.1073/pnas.0808985105 PMC2584576

[25] Willner D, Furlan M, Haynes M, Schmieder R, Angly FE, et al (2009) Metagenomic analysis of respiratory tract DNA viral communities in cystic fibrosis and non-cystic fibrosis individuals. PLoS ONE 4: e7370. 19816605 10.1371/journal.pone.0007370 PMC2756586

[26] Zhang T, Breitbart M, Lee WH, Run JQ, Wei CL, et al (2006) RNA viral community in human feces: Prevalence of plant pathogenic viruses. PLoS Biol 4: e3. 16336043 10.1371/journal.pbio.0040003 PMC1310650

[27] Breitbart M, Salamon P, Andresen B, Mahaffy JM, Segall AM, et al (2002) Genomic analysis of uncultured marine viral communities. Proc Natl Acad Sci U S A 99: 14250–14255. 12384570 10.1073/pnas.202488399 PMC137870

[28] Breitbart M, Hewson I, Felts B, Mahaffy JM, Nulton J, et al (2003) Metagenomic analyses of an uncultured viral community from human feces. J Bacteriol 185: 6220–6223. 14526037 10.1128/JB.185.20.6220-6223.2003 PMC225035

[29] Park EJ, Kim KH, Abell GCJ, Kim MS, Roh SW, et al (2011) Metagenomic analysis of the viral communities in fermented foods. Appl Environ Microbiol 77: 1284–1291. 21183634 10.1128/AEM.01859-10 PMC3067239

[30] Culley AI, Lang AS, Suttle CA (2006) Metagenomic analysis of coastal RNA virus communities. Science 312: 1795–1798. 16794078 10.1126/science.1127404

[31] Edwards RA, Rohwer F (2005) Viral metagenomics. Nat Rev Microbiol 3: 504–510. 15886693 10.1038/nrmicro1163

[32] Hino S (2002) TTV, a new human virus with single stranded circular DNA genome. Rev Med Virol 12: 151–158. 11987140 10.1002/rmv.351

[33] Kim KH, Chang HW, Nam YD, Roh SW, Kim MS, et al (2008) Amplification of uncultured single-stranded DNA viruses from rice paddy soil. Appl Environ Microbiol 74: 5975–5985. 18708511 10.1128/AEM.01275-08 PMC2565953

[34] Rosario K, Duffy S, Breitbart M (2009) Diverse circovirus-like genome architectures revealed by environmental metagenomics. J Gen Virol 90: 2418–2424. 19570956 10.1099/vir.0.012955-0

[35] Desnues C, Rodriguez-Brito B, Rayhawk S, Kelley S, Tran T, et al (2008) Biodiversity and biogeography of phages in modern stromatolites and thrombolites. Nature 452: 340–343. 18311127 10.1038/nature06735

[36] Wegley L, Breitbart M, Edwards RA, Rohwer F (2007) Metagenomic analysis of the microbial community associated with the coral *Porites astreoides.* . Environ Microbiol 9: 2707–2727. 17922755 10.1111/j.1462-2920.2007.01383.x

[37] Roux S, Enault F, Robin A, Ravet V, Personnic S, et al (2012) Assessing the diversity and specificity of two freshwater viral communities through metagenomics. PLoS ONE 7: e33641. 22432038 10.1371/journal.pone.0033641 PMC3303852

[38] Rosario K, Nilsson C, Lim YW, Ruan YJ, Breitbart M (2009) Metagenomic analysis of viruses in reclaimed water. Environ Microbiol 11: 2806–2820. 19555373 10.1111/j.1462-2920.2009.01964.x

[39] Minot S, Sinha R, Chen J, Li H, Keilbaugh SA, et al (2011) The human gut virome: Inter-individual variation and dynamic response to diet. Genome Res 21: 1616–1625. 21880779 10.1101/gr.122705.111 PMC3202279

[40] Kim MS, Park EJ, Roh SW, Bae JW (2011) Diversity and abundance of single-stranded DNA viruses in human faeces. Appl Environ Microbiol 77: 8062–8070. 21948823 10.1128/AEM.06331-11 PMC3208976

[41] Thurber RV (2009) Current insights into phage biodiversity and biogeography. Curr Opin Microbiol 12: 582–587. 19811946 10.1016/j.mib.2009.08.008

[42] Yoshida H, Ishibashi S, Watanabe Y, Inoue T, Tahara J, et al (2009) The *ABISMO* mud and water sampling ROV for surveys at 11,000 m depth. Mar Tech Soc J 43: 87–96.

[43] Middelboe M, Glud RN, Filippini M (2011) Viral abundance and activity in the deep sub-seafloor biosphere. Aquat Microb Ecol 63: 1–8.

[44] Chen F, Lu J-R, Binder BJ, Liu Y-C, Hodson RE (2001) Application of digital image analysis and flow cytometry to enumerate marine viruses stained with SYBR Gold. Appl Environ Microbiol 67: 539–545. 11157214 10.1128/AEM.67.2.539-545.2001 PMC92618

[45] Casas V, Rohwer F (2007) Phage metagenomics. Methods Enzymol 421: 259–268. 17352928 10.1016/S0076-6879(06)21020-6

[46] Altschul SF, Madden TL, Schaffer AA, Zhang JH, Zhang Z, et al (1997) Gapped BLAST and PSI-BLAST: A new generation of protein database search programs. Nucleic Acids Res 25: 3389–3402. 9254694 10.1093/nar/25.17.3389 PMC146917

[47] Huson DH, Auch AF, Qi J, Schuster SC (2007) MEGAN analysis of metagenomic data. Genome Res 17: 377–386. 17255551 10.1101/gr.5969107 PMC1800929

[48] Huson DH, Mitra S, Ruscheweyh HJ, Weber N, Schuster SC (2011) Integrative analysis of environmental sequences using MEGAN4. Genome Res 21: 1552–1560. 21690186 10.1101/gr.120618.111 PMC3166839

[49] Zhu W, Lomsadze A, Borodovsky M (2010) *Ab initio* gene identification in metagenomic sequences. Nucleic Acids Res 38: e132. 20403810 10.1093/nar/gkq275 PMC2896542

[50] Kelley DR, Liu B, Delcher AL, Pop M, Salzberg SL (2012) Gene prediction with Glimmer for metagenomic sequences augmented by classification and clustering. Nucleic Acids Res 40: e9. 22102569 10.1093/nar/gkr1067 PMC3245904

[51] Overbeek R, Begley T, Butler RM, Choudhuri JV, Chuang HY, et al (2005) The subsystems approach to genome annotation and its use in the project to annotate 1000 genomes. Nucleic Acids Res 33: 5691–5702. 16214803 10.1093/nar/gki866 PMC1251668

[52] Katoh K, Kuma K, Toh H, Miyata T (2005) MAFFT version 5: Improvement in accuracy of multiple sequence alignment. Nucleic Acids Res 33: 511–518. 15661851 10.1093/nar/gki198 PMC548345

[53] Katoh K, Toh H (2008) Recent developments in the MAFFT multiple sequence alignment program. Brief Bioinform 9: 286–298. 18372315 10.1093/bib/bbn013

[54] Saitou N, Nei M (1987) The neighbor-joining method: A new method for reconstructing phylogenetic trees. Mol Biol Evol 4: 406–425. 3447015 10.1093/oxfordjournals.molbev.a040454

[55] Tamura K, Peterson D, Peterson N, Stecher G, Nei M, et al (2011) MEGA5: Molecular evolutionary genetics analysis using maximum likelihood, evolutionary distance, and maximum parsimony methods. Mol Biol Evol 28: 2731–2739. 21546353 10.1093/molbev/msr121 PMC3203626

[56] Roux S, Faubladier M, Mahul A, Paulhe N, Bernard A, et al (2011) Metavir: A web server dedicated to virome analysis. Bioinformatics 27: 3074–3075. 21911332 10.1093/bioinformatics/btr519

[57] Martín-Cuadrado AB, Lopez-Garcia P, Alba JC, Moreira D, Monticelli L, et al (2007) Metagenomics of the deep mediterranean, a warm bathypelagic habitat. PLoS ONE 2: e914. 17878949 10.1371/journal.pone.0000914 PMC1976395

[58] Ihaka R, Gentleman R (1996) R: A language for data analysis and graphics. J Comput Graph Stat 5: 299–314.

[59] Suzuki R, Shimodaira H (2006) Pvclust: An R package for assessing the uncertainty in hierarchical clustering. Bioinformatics 22: 1540–1542. 16595560 10.1093/bioinformatics/btl117

[60] Schrenk MO, Huber JA, Edwards KJ (2010) Microbial provinces in the subseafloor. Annu Rev Mar Sci 2: 279–304. 10.1146/annurev-marine-120308-081000 21141666

[61] Morono Y, Terada T, Masui N, Inagaki F (2009) Discriminative detection and enumeration of microbial life in marine subsurface sediments. ISME J 3: 503–511. 19212428 10.1038/ismej.2009.1

[62] Seiter K, Hensen C, Schröter J, Zabel M (2004) Organic carbon content in surface sediments–defining regional provinces. Deep-Sea Res I 51: 2001–2026.

[63] Breitbart M, Felts B, Kelley S, Mahaffy JM, Nulton J, et al (2004) Diversity and population structure of a near-shore marine-sediment viral community. Proc Biol Sci 271: 565–574. 15156913 10.1098/rspb.2003.2628 PMC1691639

[64] Sullivan MB, Krastins B, Hughes JL, Kelly L, Chase M, et al (2009) The genome and structural proteome of an ocean siphovirus: A new window into the cyanobacterial ‘mobilome’. Environ Microbiol 11: 2935–2951. 19840100 10.1111/j.1462-2920.2009.02081.x PMC2784084

[65] Nagasaki K, Tomaru Y, Takao Y, Nishida K, Shirai Y, et al (2005) Previously unknown virus infects marine diatom. Appl Environ Microbiol 71: 3528–3535. 16000758 10.1128/AEM.71.7.3528-3535.2005 PMC1169059

[66] Tomaru Y, Shirai Y, Suzuki H, Nagumo T, Nagasaki K (2008) Isolation and characterization of a new single-stranded DNA virus infecting the cosmopolitan marine diatom *Chaetoceros debilis* . Aqua Microbial Ecol 50: 103–112.

[67] Tomaru Y, Shirai Y, Toyoda K, Nagasaki K (2011) Isolation and characterisation of a single-stranded DNA virus infecting the marine planktonic diatom *Chaetoceros tenuissimus* Meunier. Aquat Microb Ecol 64: 175–184. 10.1128/AEM.00509-08 PMC2446501 18469125

[68] Tomaru Y, Takao Y, Suzuki H, Nagumo T, Koike K, et al (2011) Isolation and characterization of a single-stranded DNA virus infecting *Chaetoceros lorenzianus* Grunow. Appl Environ Microbiol 77: 5285–5293. 21666026 10.1128/AEM.00202-11 PMC3147440

[69] Holmfeldt K, Odić D, Sullivan MB, Middelboe M, Riemann L (2012) Cultivated single-stranded DNA phages that infect marine *Bacteroidetes* prove difficult to detect with DNA-binding stains. Appl Environ Microbiol 78: 892–894. 22138992 10.1128/AEM.06580-11 PMC3264134

[70] Tucker KP, Parsons R, Symonds EM, Breitbart M (2010) Diversity and distribution of single-stranded DNA phages in the North Atlantic Ocean. ISME J 5: 822–830. 21124487 10.1038/ismej.2010.188 PMC3105770

[71] Yoon HS, Price DC, Stepanauskas R, Rajah VD, Sieracki ME, et al (2011) Single-cell genomics reveals organismal interactions in uncultivated marine protists. Science 332: 714–717. 21551060 10.1126/science.1203163

[72] Gibbs MJ, Smeianov VV, Steele JL, Upcroft P, Efimov BA (2006) Two families of rep-like genes that probably originated by interspecies recombination are represented in viral, plasmid, bacterial, and parasitic protozoan genomes. Mol Biol Evol 23: 1097–1100. 16531508 10.1093/molbev/msj122

[73] Liu H, Fu Y, Li B, Yu X, Xie J, et al (2011) Widespread horizontal gene transfer from circular single-stranded DNA viruses to eukaryotic genomes. BMC Evol Biol 11: 276. 21943216 10.1186/1471-2148-11-276 PMC3198968

[74] Martin DP, Biagini P, Lefeuvre P, Golden M, Roumagnac P, et al (2011) Recombination in eukaryotic single stranded DNA viruses. Viruses 3: 1699–1738. 21994803 10.3390/v3091699 PMC3187698

[75] Mann NH, Cook A, Millard A, Bailey S, Clokie M (2003) Marine ecosystems: Bacterial photosynthesis genes in a virus. Nature 424: 741. 10.1038/424741a 12917674

[76] Duhaime MB, Deng L, Poulos BT, Sullivan MB (2012) Towards quantitative metagenomics of wild viruses and other ultra-low concentration DNA samples: A rigorous assessment and optimization of the linker amplification method. Environ Microbiol 14: 2526–2537. 22713159 10.1111/j.1462-2920.2012.02791.x PMC3466414

[77] Duhaime MB, Sullivan MB (2012) Ocean viruses: Rigorously evaluating the metagenomic sample-to-sequence pipeline. Virology 434: 181–186. 23084423 10.1016/j.virol.2012.09.036

[78] Hurwitz BL, Deng L, Poulos BT, Sullivan MB (2012) Evaluation of methods to concentrate and purify ocean virus communities through comparative, replicated metagenomics. Environ Microbiol doi:10.1111/j.1462-2920.2012.02836.x. 10.1111/j.1462-2920.2012.02836.x PMC3655615 22845467

[79] Andrews-Pfannkoch C, Fadrosh DW, Thorpe J, Williamson SJ (2010) Hydroxyapatite-mediated separation of double-stranded DNA, single-stranded DNA, and RNA genomes from natural viral assemblages. Appl Environ Microbiol 76: 5039–5045. 20543058 10.1128/AEM.00204-10 PMC2916501

