# Supplementary figures and images for: Metagenomic Analysis of Viral Communities in (Hado)Pelagic Sediments
Source: PLoS One. 2013 Feb 27;8(2):e57271. doi: 10.1371/journal.pone.0057271 (PMC3584133; doi:10.1371/journal.pone.0057271)

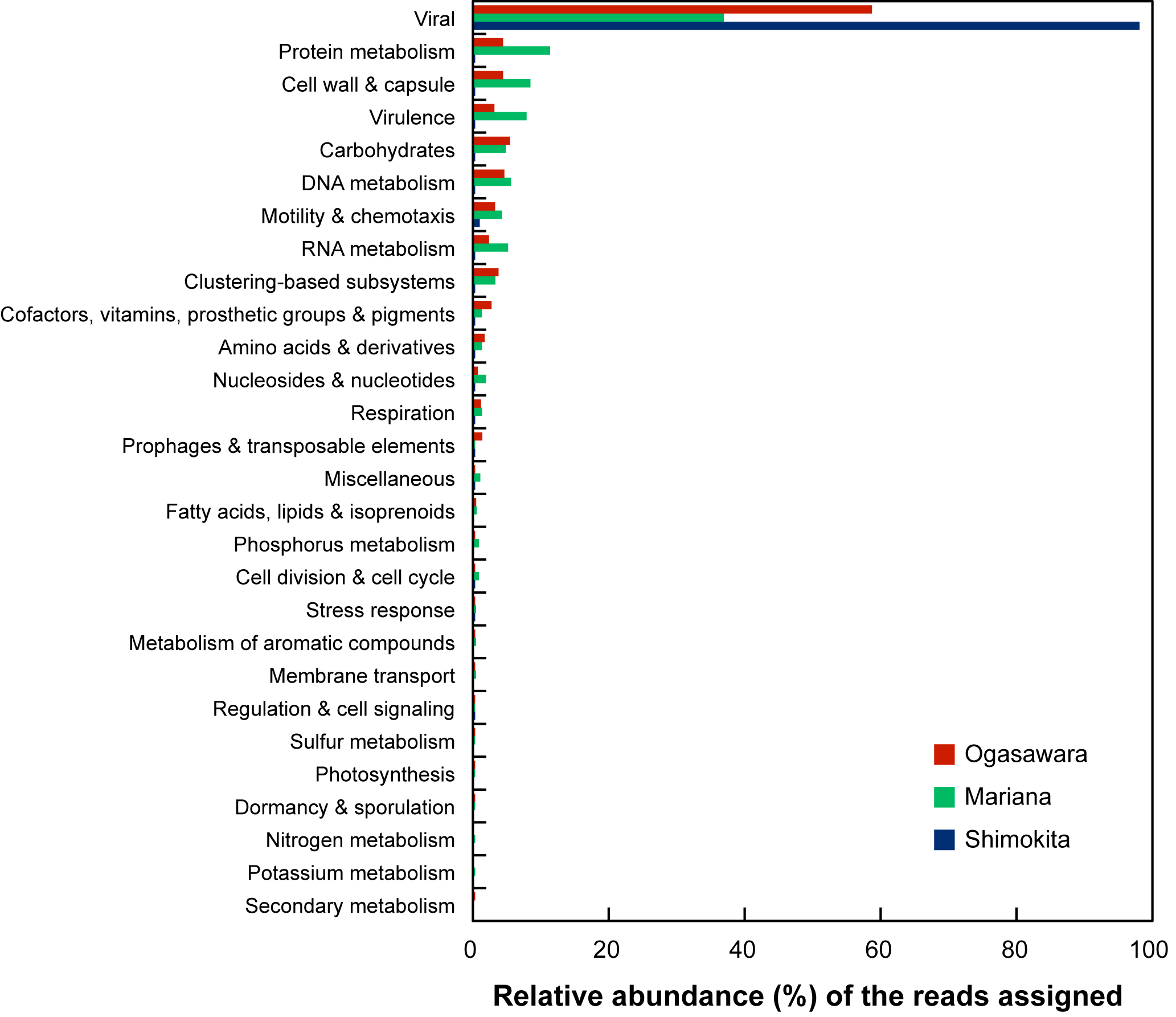

Supplement: Figure S1 — Profiles of the function categories for the genes predicted from three deep-sea shallow subseafloor sedimentary viromes. The relative abundance of the constituent sequence reads of the virome genes assigned to SEED subsystems [51] with significance (E-value <10−3 in BLASTp) is shown. (TIF) [file pone.0057271.s001.tif]

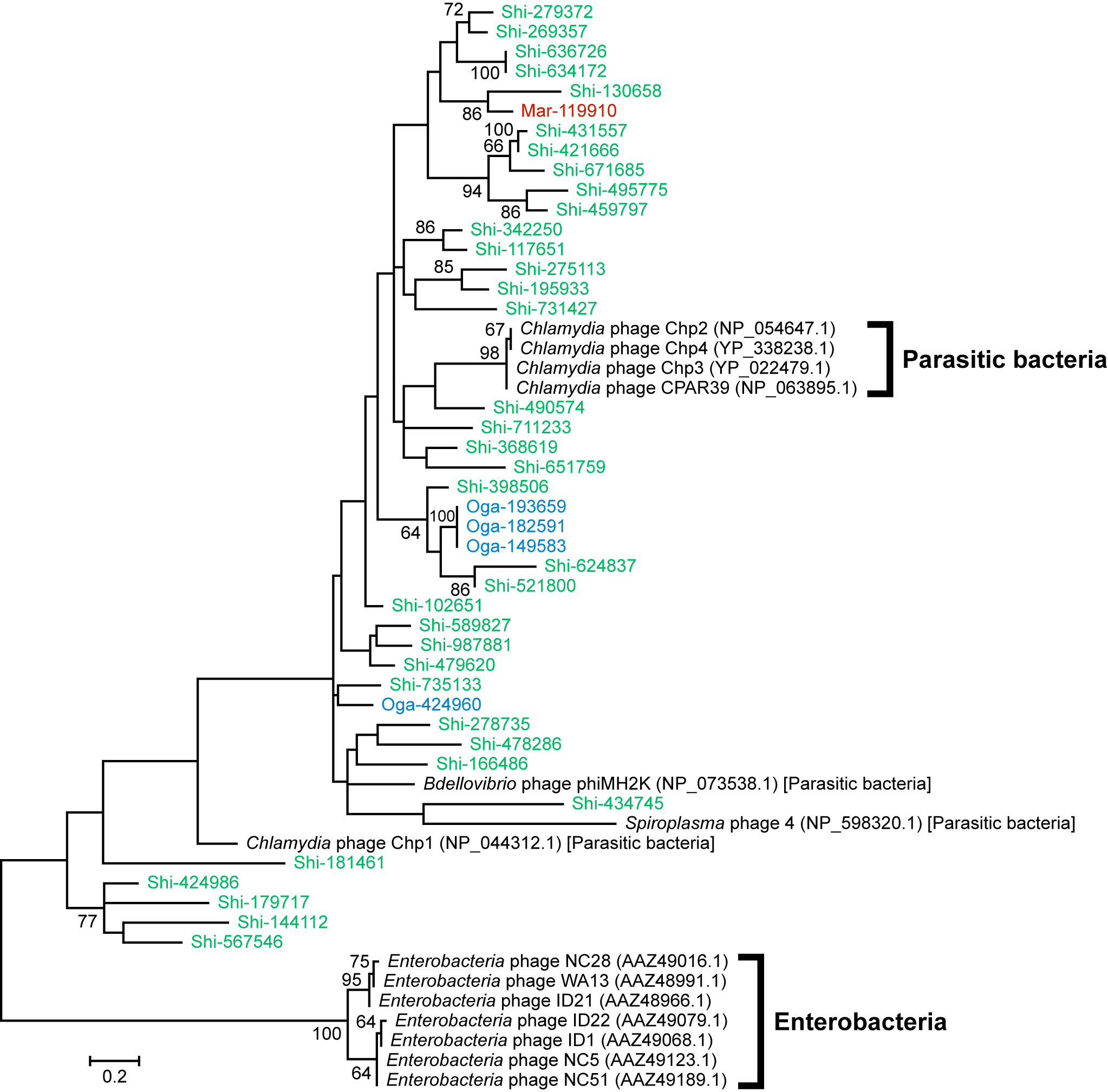

Supplement: Figure S2 — Maximum-likelihood tree of the 58 amino acid sequences of the major capsid protein (VP1 marker for Microviridae ) from the contigs in the virome libraries, as represented by a tree gallery (the 50 ‘best’ trees) with the MetaVir workflow [56] . The virome sequences from the Ogasawara (OG), Mariana (MA), and Shimokita (SH) libraries are highlighted in blue, red, and green, respectively. The numbers in parentheses indicate the DDBJ/EMBL/GenBank accession numbers for the sequences. Only bootstrap values of >50% are indicated at the nodes of the tree. (TIF) [file pone.0057271.s002.tif]

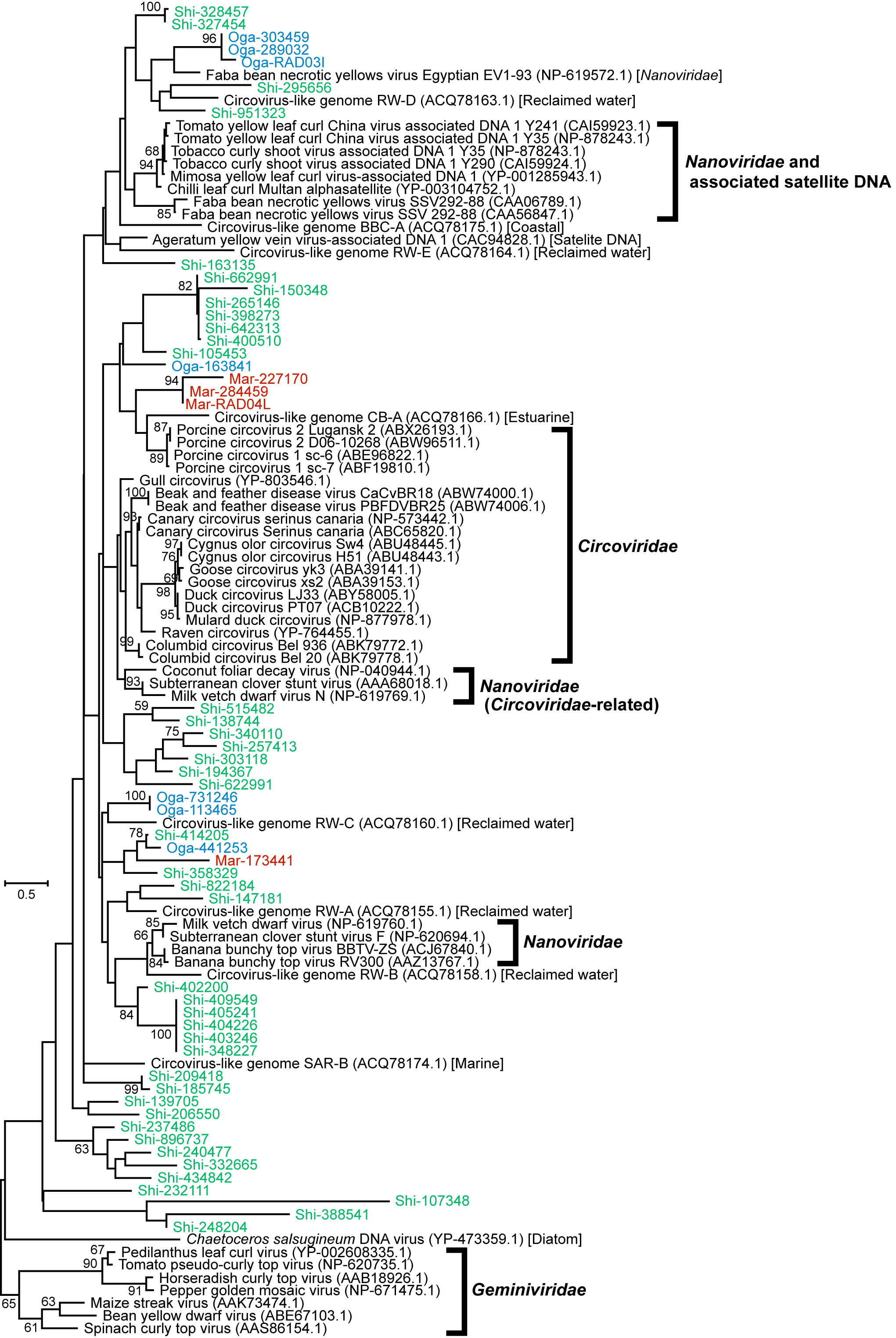

Supplement: Figure S3 — The neighbor-joining phylogenetic tree of the 52 amino acid sequences of the replication protein (Rep marker for the Circoviridae − Nanoviridae − Geminiviridae group) from the contigs in the virome libraries as represented by a tree gallery (the 50 ‘best’ trees) with the MetaVir workflow [56] . The virome sequences from the Ogasawara (OG), Mariana (MA), and Shimokita (SH) libraries are highlighted in blue, red, and green, respectively. The numbers in parentheses indicate the DDBJ/EMBL/GenBank accession numbers for the sequences. Only bootstrap values of >50% are indicated at the nodes of the tree. (TIF) [file pone.0057271.s003.tif]
