# Supplementary material for: Metagenomic Analysis of Viral Communities in (Hado)Pelagic Sediments
Source: PLoS One. 2013 Feb 27;8(2):e57271. doi: 10.1371/journal.pone.0057271 (PMC3584133; doi:10.1371/journal.pone.0057271)
Supplement: Table S1 — Representative viral genetic markers used in the MetaVir workflow [56] . (DOC) [file pone.0057271.s004.doc]

**Table S1. Representative viral genetic markers used in the MetaVir workflow [56].**

| **Marker** | **Viral group** | **Function** | **PFAM ID** | **Number of reference sequences** | **Reference** |
| --- | --- | --- | --- | --- | --- |
| Rep | *Circoviridae*−*Nanoviridae*−*Geminiviridae* | Replication protein | PF02407, *Circo*-*Nano*; PF00799, *Gemini* | 53 | [34] |
| VP1 | *Microviridae* | Major capsid protein | PF02305 | 50 | [35] |
| G20 | T4-like Bacteriophages | Capsid assembly protein | PF07230 | 204 | [90] |
| GP23 | T4-like Bacteriophages | Major capsid protein | PF07068 | 24 | [91] |
| T7gp17 | Podoviridae | Phage tail fiber | PF03906 | 36 | − |
| PsbA | Cultivated cyanophages | Photosynthetic reaction center protein | PF00124 | 99 | [92] |
| TerL | *Caudovirales* | Terminase Large subunit | PF03237 | 198 | [64] |
| PolB | dsDNA viruses, bacteria, and eukaryotes | DNA Polymerase family B | PF00136 | 34 | [93] |
| MCP | Large eukaryotic dsDNA viruses (*Phycodnaviridae*, *Iridoviridae*, *Asfaviridae*, *Ascoviridae*, and *Mimiviridae*) | Major capsid protein | PF04451 | 97 | [94] |
| RdRP | RNA viruses | RNA-dependent RNA polymerase | PF00680 | 111 | [30] |

The reference sequences corresponding to each marker gene were taken from the PFAM database [83].

**References**

(References cited in the main paper are not repeated here)

1. Dorigo U, Jacquet S, Humbert J (2004) Cyanophage diversity, inferred from *g20* gene analyses, in the largest natural lake in France, Lake Bourget. Appl Environ Microbiol 70: 1017–1022.
2. Comeau AM, Krisch HM (2008) The capsid of the T4 phage superfamily: The evolution, diversity, and structure of some of the most prevalent proteins in the biosphere. Mol Biol Evol 25: 1321–1332.
3. Chénard C, Suttle CA (2008) Phylogenetic diversity of sequences of cyanophage photosynthetic gene *psbA* in marine and freshwaters. Appl Environ Microbiol 74: 5317–5324.
4. Monier A, Claverie J, Ogata H (2008) Taxonomic distribution of large DNA viruses in the sea. Genome Biol 9: R106.
5. Rowe JM, Fabre M-F, Gobena D, Wilson WH, Wilhelm SW (2011) Application of the major capsid protein as a marker of the phylogenetic diversity of *Emiliania huxleyi* viruses. FEMS Microbiol Ecol 76: 373–380.
