# Supplementary material for: Metagenomic Analysis of Viral Communities in (Hado)Pelagic Sediments
Source: PLoS One. 2013 Feb 27;8(2):e57271. doi: 10.1371/journal.pone.0057271 (PMC3584133; doi:10.1371/journal.pone.0057271)
Supplement: Table S2 — Viral abundance in the (hado)pelagic surface sediments (down to 30 cmbsf) from the Ogasawara, Mariana, and Shimokita locations. (DOC) [file pone.0057271.s005.doc]

**Table S2.** Viral abundance in the (hado)pelagic surface sediments (down to 30 cmbsf) from the Ogasawara, Mariana, and Shimokita locations.

| **Sampling site** | **Sediment layer (cmbsf)** | **Viral abundance (viruses/cm^3^)** |
| --- | --- | --- |
| Ogasawara (OG) | 0−10 | 5.8 × 10^7^ |
|  | 10−20 | 6.2 × 10^7^ |
|  | 20−30 | 6.6 × 10^7^ |
| Mariana (MA) | 0−5 | 5.3 × 10^7^ |
|  | 5−10 | 1.2 × 10^7^ |
|  | 10−15 | 1.8 × 10^7^ |
|  | 15−20 | 8.1 × 10^6^ |
|  | 20−25 | 2.4 × 10^6^ |
|  | 25−30 | 4.2 × 10^6^ |
| Shimokita (SH) | 0−2 | 1.2 × 10^11^ |
|  | 2−5 | 1.8 × 10^11^ |
|  | 5−10 | 7.6 × 10^10^ |
|  | 10−15 | 9.9 × 10^7^ |
|  | 15−20 | 1.1 × 10^8^ |
|  | 20−25 | 2.0 × 10^8^ |
|  | 25−30 | 2.7 × 10^8^ |
