# Supplementary material for: Metagenomic Analysis of Viral Communities in (Hado)Pelagic Sediments
Source: PLoS One. 2013 Feb 27;8(2):e57271. doi: 10.1371/journal.pone.0057271 (PMC3584133; doi:10.1371/journal.pone.0057271)
Supplement: Materials and Methods S1 — Prokaryotic 16S rRNA gene clone library analysis. (PDF) [file pone.0057271.s006.pdf]

Materials and Methods S1

Prokaryotic 16S rRNA gene clone library analysis

Bacterial and archaeal 16S rRNA genes were simultaneously amplified from DNA extracts by PCR with LA Taq polymerase and GC buffer (TaKaRa Bio, Otsu, Japan). The oligonucleotide primers used were mixtures of various derivatives of previously designed 530F and 907R primers as described previously [80]. Thermal cycling was performed under the following conditions: an initial preheating for 5 min at 96°C; 25−40 cycles of denaturation at 96 °C for 25 sec, annealing at 50 °C for 45 sec, and extension at 72 °C for 30 sec; a final extension at 72°C for 7 min. The PCR cycle numbers represent the minimum cycle numbers that provided enough amplified products for cloning based on the preliminary PCR amplification experiments using the same templates. The amplified rRNA gene products from several separate reactions using the lowest number of thermal cycles were pooled and purified as described previously [81]. Cloning and sequencing were performed according to the procedure described by Takai et al. [81]. A single strand with the cloned rRNA gene fragment (approximately 450 bp) was sequenced using the M13M4 oligonucleotide primer. The sequence similarity analysis of the obtained sequences was performed with GENETYX-MAC software, ver. 12 (GENETYX Corporation, Tokyo, Japan), to identify representative sequences. The sequences with >97% similarity were identified as the same phylotype. The sequences of the representative phylotypes were classified based on the similarity search results obtained by using the SINA alignment service (http://www.arb-silva.de/aligner/) [82] with reference sequences in SILVA [83].

References

(References cited in the main paper are not repeated here)

80. Nunoura T, Takaki Y, Kazama H, Hirai M, Ashi J, et al. (2012) Microbial diversity in deep-sea methane seep sediments presented by SSU rRNA gene tag sequencing. Microbes Environ 27: 382–90.
81. Takai K, Moser DP, DeFlaun M, Onstott TC, Fredrickson JK (2001) Archaeal diversity in waters from deep South African gold mines. Appl Environ Microbiol 67: 5750–5760.
82. Pruesse E, Peplies J, Glöckner FO (2012) SINA: Accurate high throughput multiple sequence alignment of ribosomal RNA genes. Bioinformatics 28: 1823–1829.
83. Pruesse E, Quast C, Knittel K, Fuchs BM, Ludwig W, et al. (2007) SILVA: A comprehensive online resource for quality checked and aligned ribosomal RNA sequence data compatible with ARB. Nucleic Acids Res 35: 7188–7196.
