# Supplementary material for: Metagenomic Analysis of Viral Communities in (Hado)Pelagic Sediments
Source: PLoS One. 2013 Feb 27;8(2):e57271. doi: 10.1371/journal.pone.0057271 (PMC3584133; doi:10.1371/journal.pone.0057271)
Supplement: Materials and Methods S2 — Online phylogenetic analysis of the ssDNA viral sequences with MetaVir. (PDF) [file pone.0057271.s007.pdf]

Materials and Methods S2

Online phylogenetic analysis of the ssDNA viral sequences with MetaVir

This section describes the online phylogenetic analysis with MetaVir (http://metavir-meb.univ-bpclermont.fr/) [56]. The sequence reads with significant similarity (E-values >10^{-3} in BLASTx) to the ssDNA viral genetic markers (Table S1) were obtained from each virome library, assembled using Cap3 [84] (98% identity in 35 bp), and used to construct phylogenetic trees alongside reference sequences taken from the protein family (PFAM) database [85,86] (Table 3). These amino acid sequences from the contigs were aligned against the reference alignment via a HMM profile using HMMER [87]. Because most of the ssDNA viral sequences from the libraries did not cover the entire gene sequence, different phylogenetic trees were generated for conserved regions of each viral gene. To generate trees containing as many viral sequences from the libraries as possible, alignment bounds for each viral sequence were collected and used to define multiple subalignments. Ambiguous regions in alignments were removed using Gblocks [88], and phylogenetic trees with 100 bootstraps were constructed using PhyML [89]. Consequently, a tree gallery containing up to 50 of the 'best' trees was systematically constructed.

References

(References cited in the main paper are not repeated here)

84. Huang X, Madan A (1999) CAP3: A DNA sequence assembly program. Genome Res 9: 868–877.

85. Finn RD, Mistry J, Schuster-Bockler B, Griffiths-Jones S, Hollich V, et al. (2006) Pfam: clans, web tools and services. Nucleic Acids Res 34: D247–251.
86. Finn RD, Mistry J, Tate J, Coggill P, Heger A, et al. (2010) The Pfam protein families database. Nucleic Acids Res 38: D211–222.
87. Eddy SR (1998) Profile hidden Markov models. Bioinformatics 14: 755–763.
88. Talavera G, Castresana J (2007) Improvement of phylogenies after removing divergent and ambiguously aligned blocks from protein sequence alignments. Syst Biol 56: 564–577.
89. Guindon S, Delsuc F, Dufayard JF, Gascuel O (2009) Estimating maximum likelihood phylogenies with PhyML. Methods Mol Biol 537: 113–37.
